# Supplementary material for: The Causal Relationship between the Morning Chronotype and the Gut Microbiota: A Bidirectional Two-Sample Mendelian Randomization Study
Source: Nutrients. 2023 Dec 22;16(1):46. doi: 10.3390/nu16010046 (PMC10780629; doi:10.3390/nu16010046)
Supplement: Supplementary file 1 [file nutrients-16-00046-s001.zip › nutrients-2751257-supplementary figures.pdf]

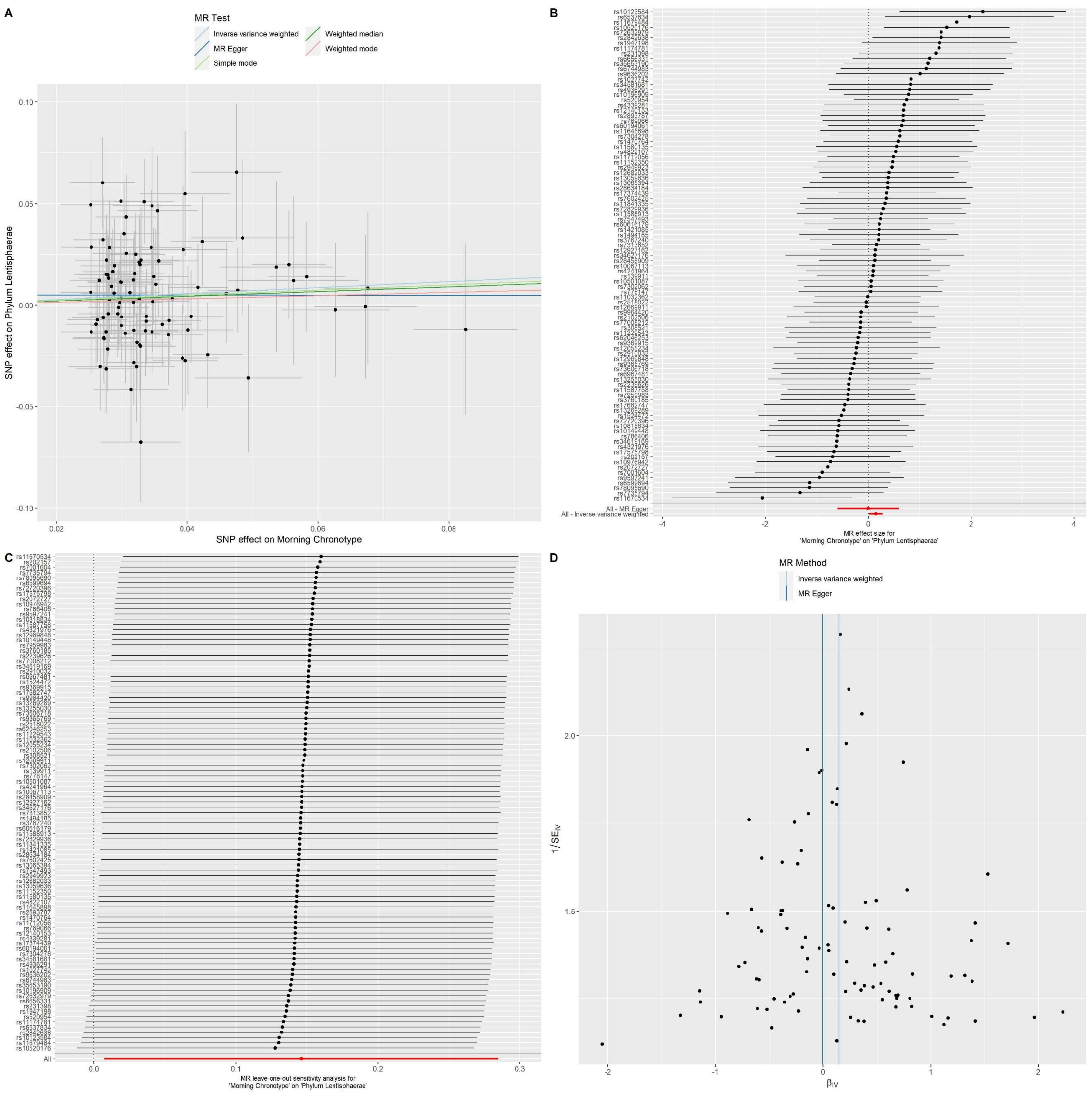

Figure S1. Mendelian randomization plots for the relationship of morning chronotype with Phylum *Lentisphaerae*

Note: A, Scatterplot of SNP effects on Phylum *Lentisphaerae* with the slope of each line corresponding to estimated MR effect (Inverse Variance Weighted, Weight Median, MR-Egger, Weighted Mode, and Simple Mode methods); B, Forest plot of individual and combined SNP MR-estimated effects sizes for relative Phylum *Lentisphaerae*; C, The leave-one-out plot visualized how the causal estimates (point with horizontal line) for the effect of morning chronotype on Phylum *Lentisphaerae* were influenced by the removal of single variant; D, Funnel plot assessing heterogeneity. Blue line represents the inverse-variance weighted estimate, and dark blue line represents the MR-Egger estimate.

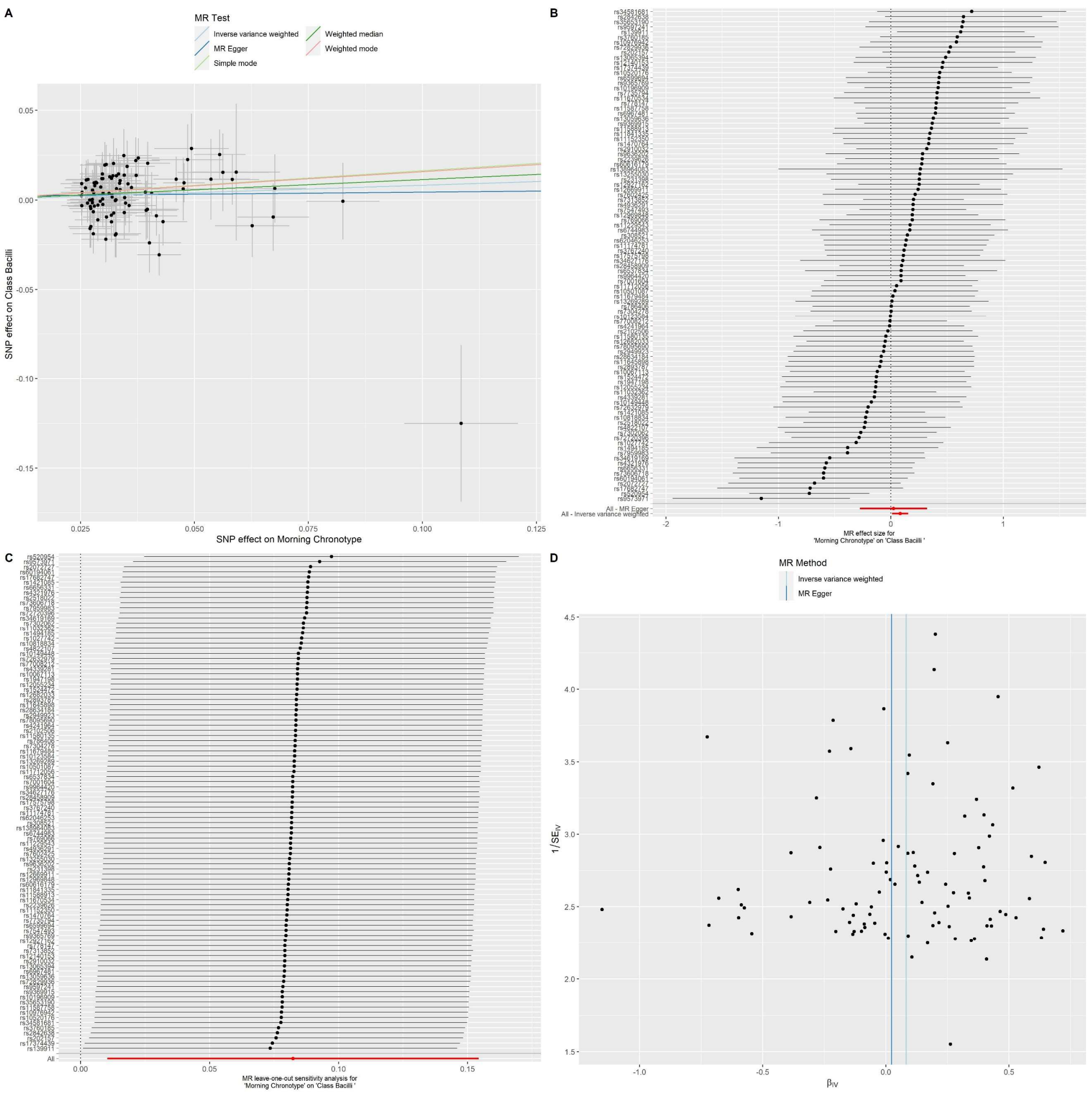

Figure S2. Mendelian randomization plots for the relationship of morning chronotype with Class *Bacilli*

Note: A, Scatterplot of SNP effects on Class *Bacilli* with the slope of each line corresponding to estimated MR effect (Inverse Variance Weighted, Weight Median, MR-Egger, Weighted Mode, and Simple Mode methods); B, Forest plot of individual and combined SNP MR-estimated effects sizes for relative Class *Bacilli*; C, The leave-one-out plot visualized how the causal estimates (point with horizontal line) for the effect of morning chronotype on Class *Bacilli* were influenced by the removal of single variant; D, Funnel plot assessing heterogeneity. Blue line represents the inverse-variance weighted estimate, and dark blue line represents the MR-Egger estimate.

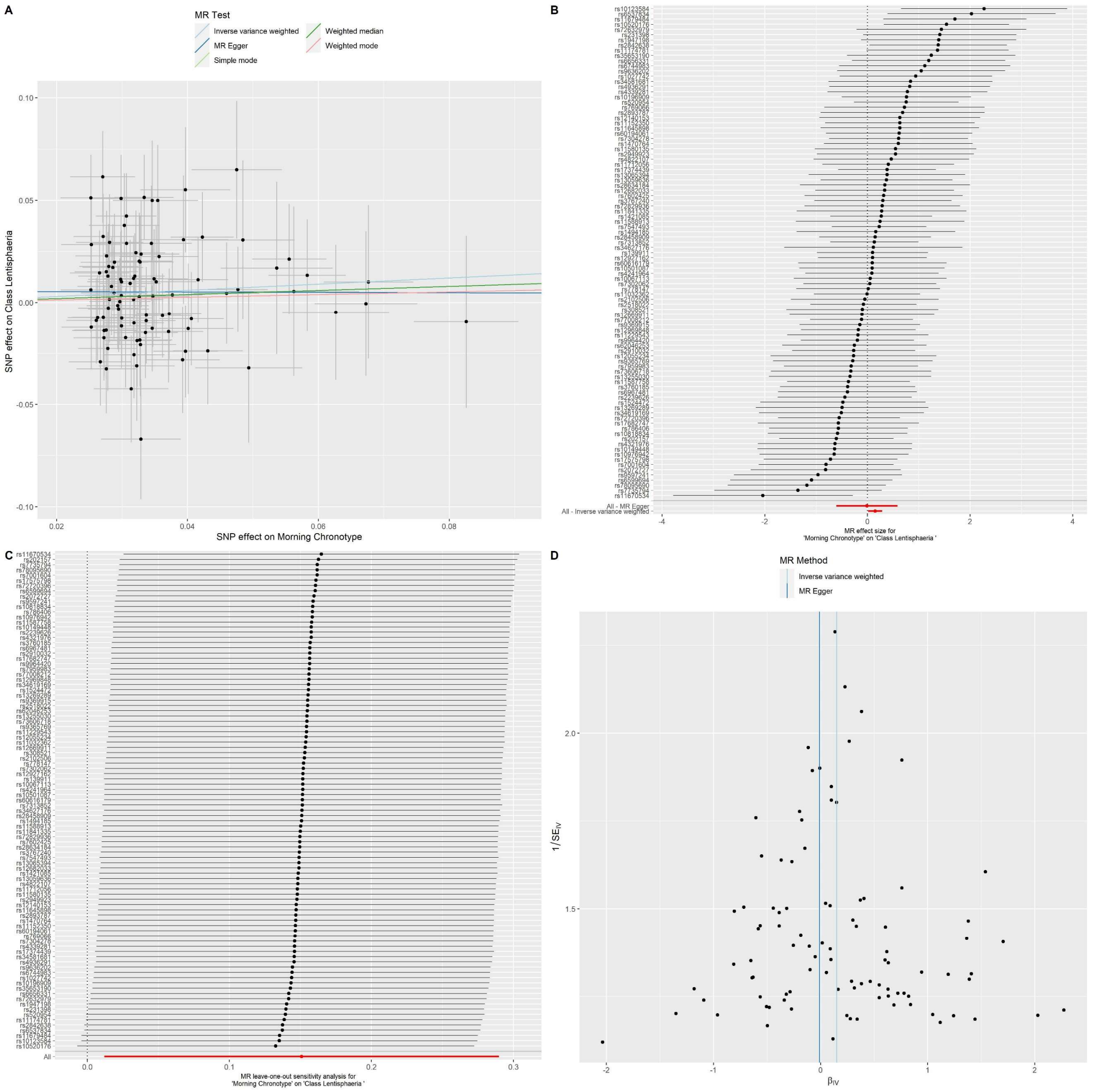

Figure S3. Mendelian randomization plots for the relationship of morning chronotype with Class *Lentisphaeria*

Note: A, Scatterplot of SNP effects on Class *Lentisphaeria* with the slope of each line corresponding to estimated MR effect (Inverse Variance Weighted, Weight Median, MR-Egger, Weighted Mode, and Simple Mode methods); B, Forest plot of individual and combined SNP MR-estimated effects sizes for relative Class *Lentisphaeria*; C, The leave-one-out plot visualized how the causal estimates (point with horizontal line) for the effect of morning chronotype on Class *Lentisphaeria* were influenced by the removal of single variant; D, Funnel plot assessing heterogeneity. Blue line represents the inverse-variance weighted estimate, and dark blue line represents the MR-Egger estimate.

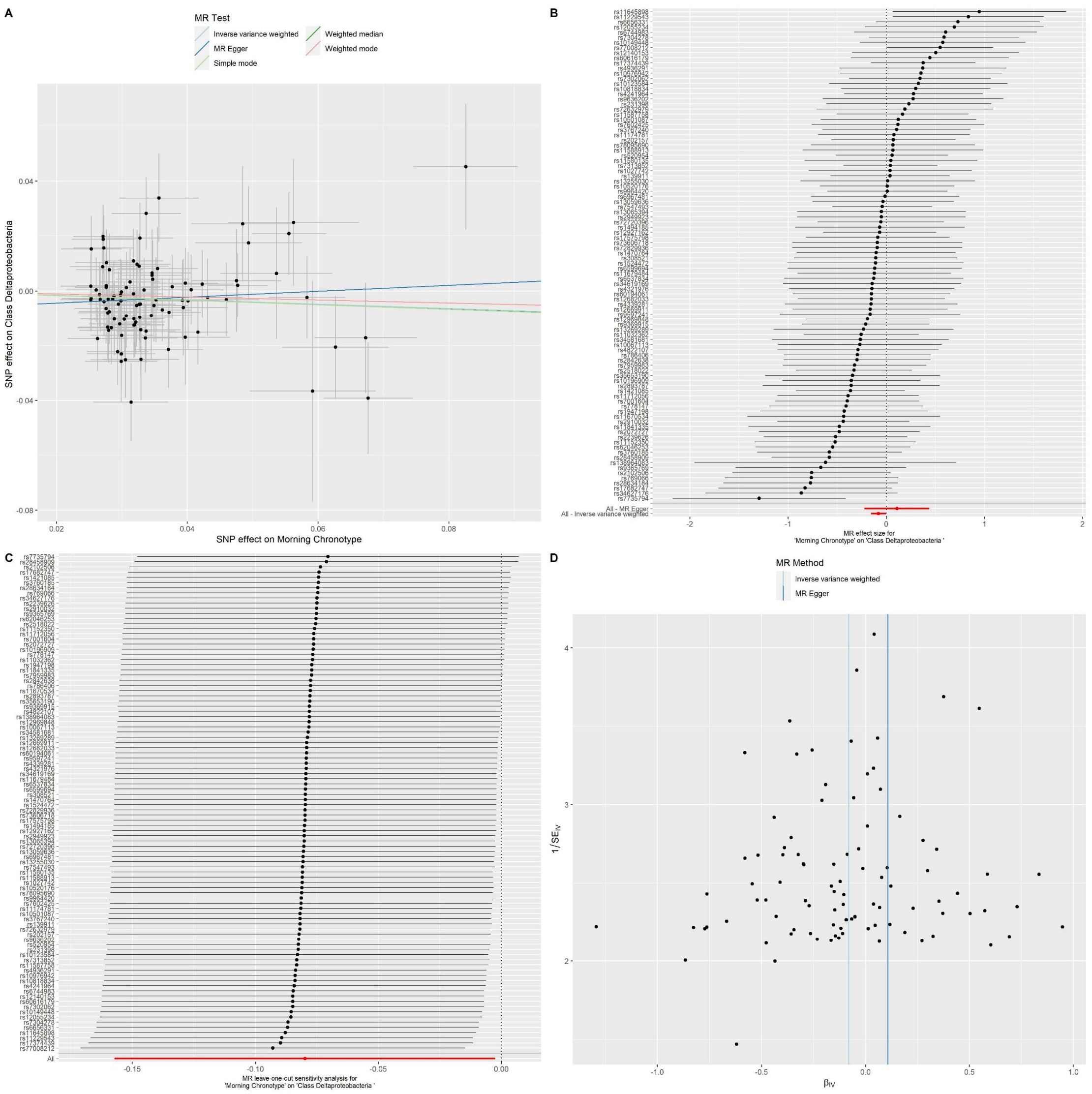

Figure S4. Mendelian randomization plots for the relationship of morning chronotype with Class *Deltaproteobacteria*

Note: A, Scatterplot of SNP effects on Class *Deltaproteobacteria* with the slope of each line corresponding to estimated MR effect (Inverse Variance Weighted, Weight Median, MR-Egger, Weighted Mode, and Simple Mode methods); B, Forest plot of individual and combined SNP MR-estimated effects sizes for relative Class *Deltaproteobacteria*; C, The leave-one-out plot visualized how the causal estimates (point with horizontal line) for the effect of morning chronotype on Class *Deltaproteobacteria* were influenced by the removal of single variant; D, Funnel plot assessing heterogeneity. Blue line represents the inverse-variance weighted estimate, and dark blue line represents the MR-Egger estimate.

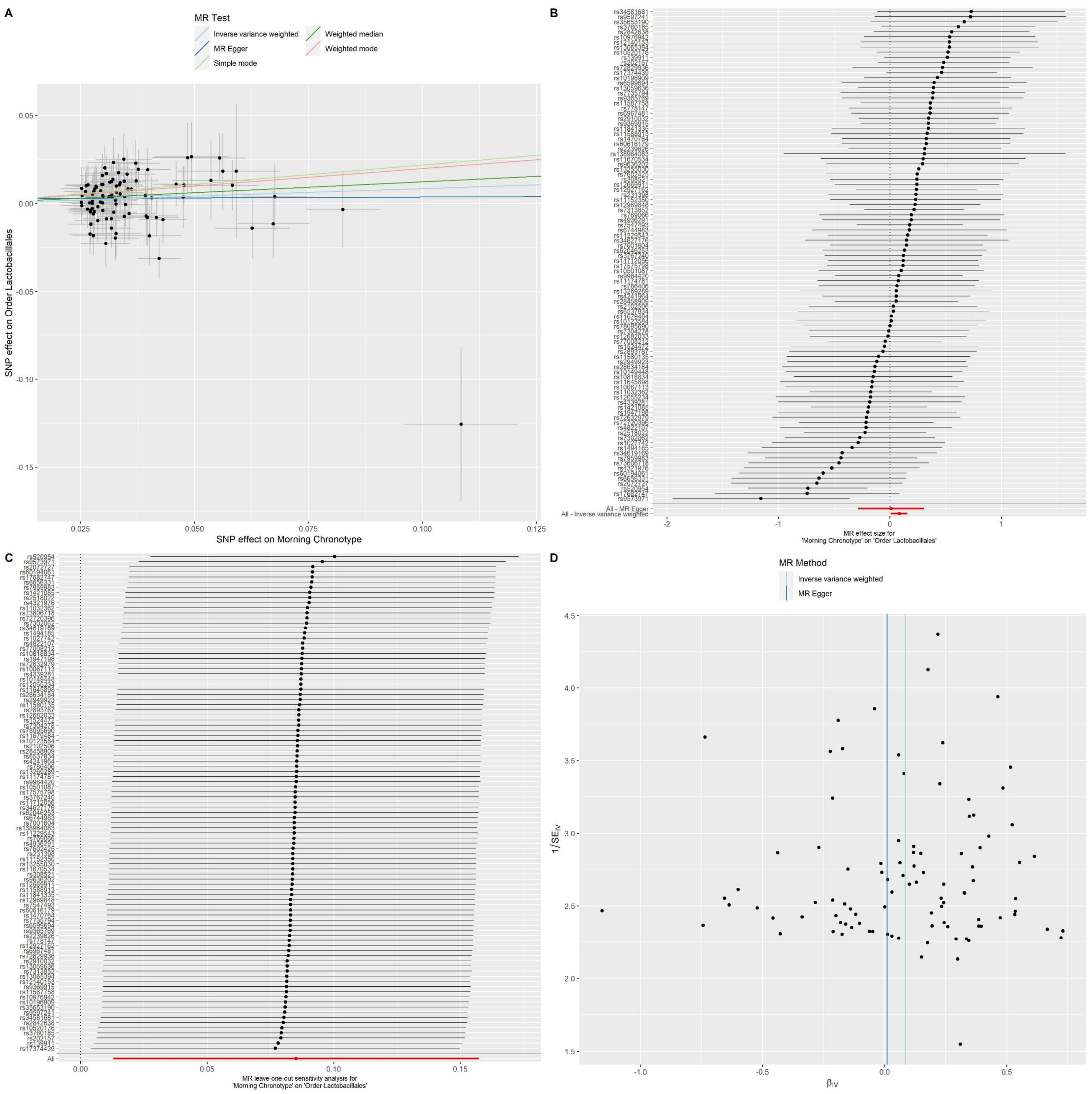

Figure S5. Mendelian randomization plots for the relationship of morning chronotype with Order *Lactobacillales*

Note: A, Scatterplot of SNP effects on Order *Lactobacillales* with the slope of each line corresponding to estimated MR effect(Inverse Variance Weighted, Weight Median, MR-Egger, Weighted Mode, and Simple Mode methods); B, Forest plot of individual and combined SNP MR-estimated effects sizes for relative Order *Lactobacillales*; C, The leave-one-out plot visualized how the causal estimates (point with horizontal line) for the effect of morning chronotype on Order *Lactobacillales* were influenced by the removal of single variant; D, Funnel plot assessing heterogeneity. Blue line represents the inverse-variance weighted estimate, and dark blue line represents the MR-Egger estimate.

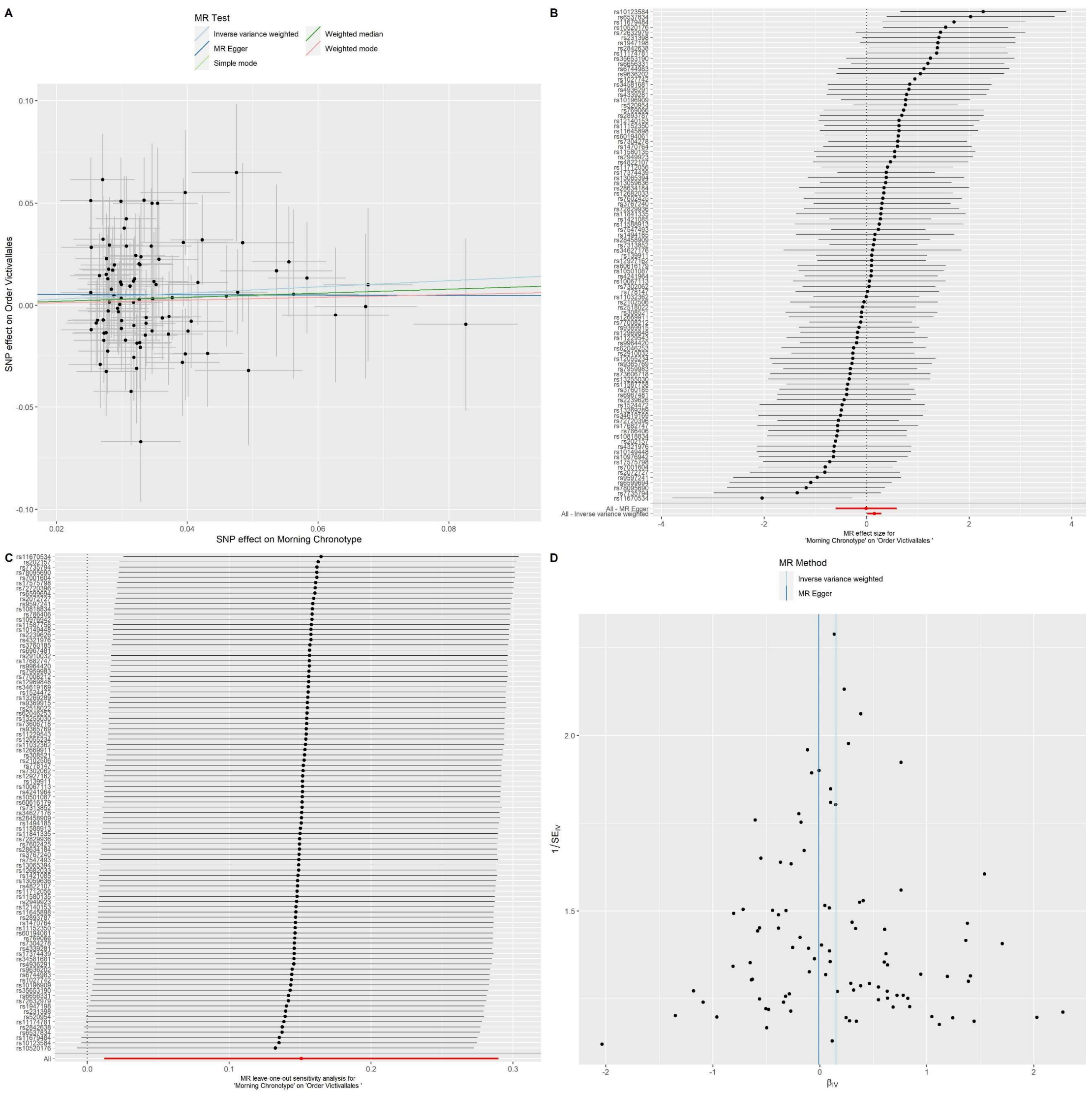

Figure S6. Mendelian randomization plots for the relationship of morning chronotype with Order *Victivallales*

Note: A, Scatterplot of SNP effects on Order *Victivallales* with the slope of each line corresponding to estimated MR effect (Inverse Variance Weighted, Weight Median, MR-Egger, Weighted Mode, and Simple Mode methods); B, Forest plot of individual and combined SNP MR-estimated effects sizes for relative Order *Victivallales*; C, The leave-one-out plot visualized how the causal estimates (point with horizontal line) for the effect of morning chronotype on Order *Victivallales* were influenced by the removal of single variant; D, Funnel plot assessing heterogeneity. Blue line represents the inverse-variance weighted estimate, and dark blue line represents the MR-Egger estimate.

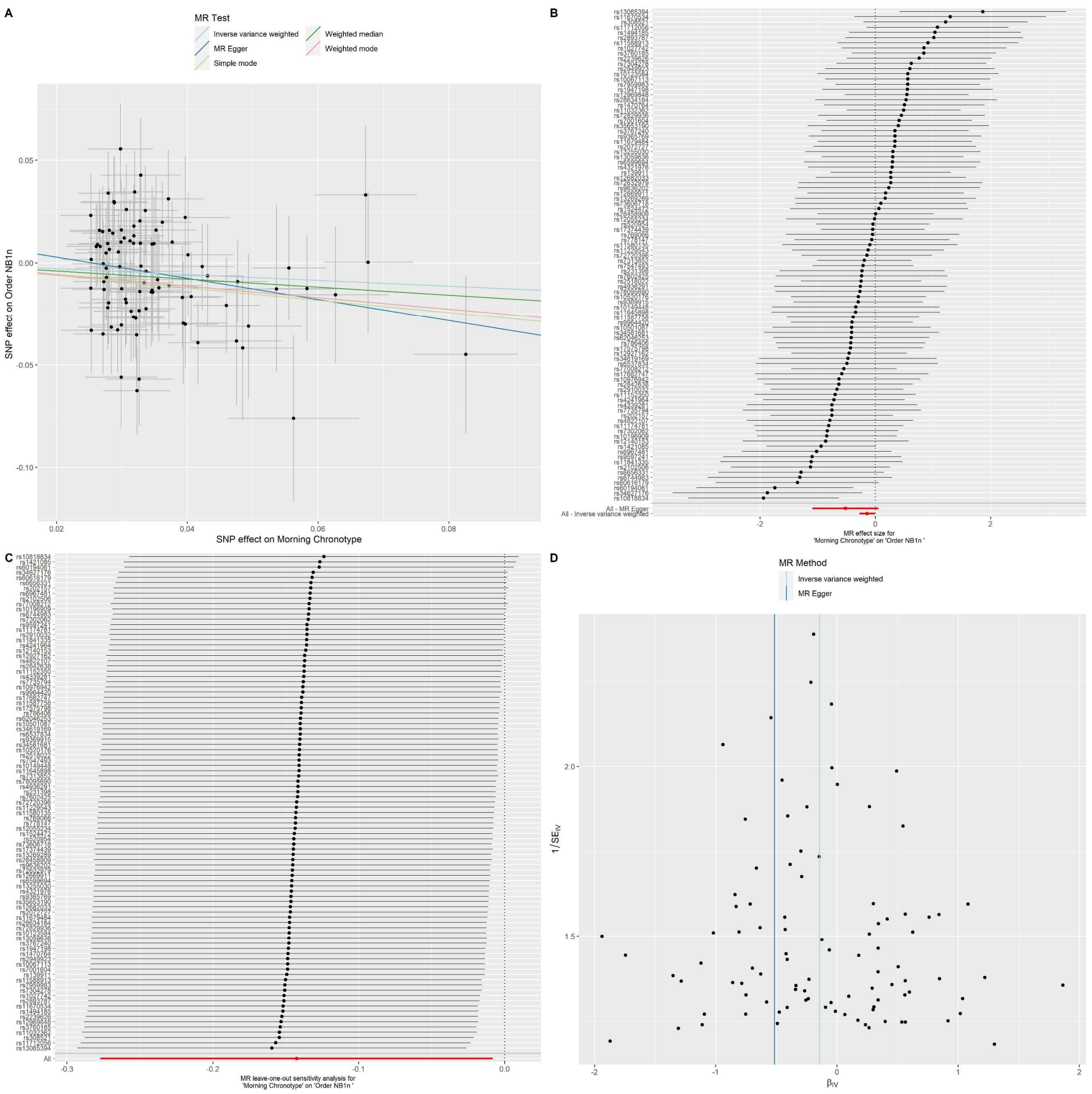

Figure S7. Mendelian randomization plots for the relationship of morning chronotype with Order *NB1n*

Note: A, Scatterplot of SNP effects on Order *NB1n* with the slope of each line corresponding to estimated MR effect (Inverse Variance Weighted, Weight Median, MR-Egger, Weighted Mode, and Simple Mode methods); B, Forest plot of individual and combined SNP MR-estimated effects sizes for relative Order *NB1n*; C, The leave-one-out plot visualized how the causal estimates (point with horizontal line) for the effect of morning chronotype on Order *NB1n* were influenced by the removal of single variant; D, Funnel plot assessing heterogeneity. Blue line represents the inverse-variance weighted estimate, and dark blue line represents the MR-Egger estimate.

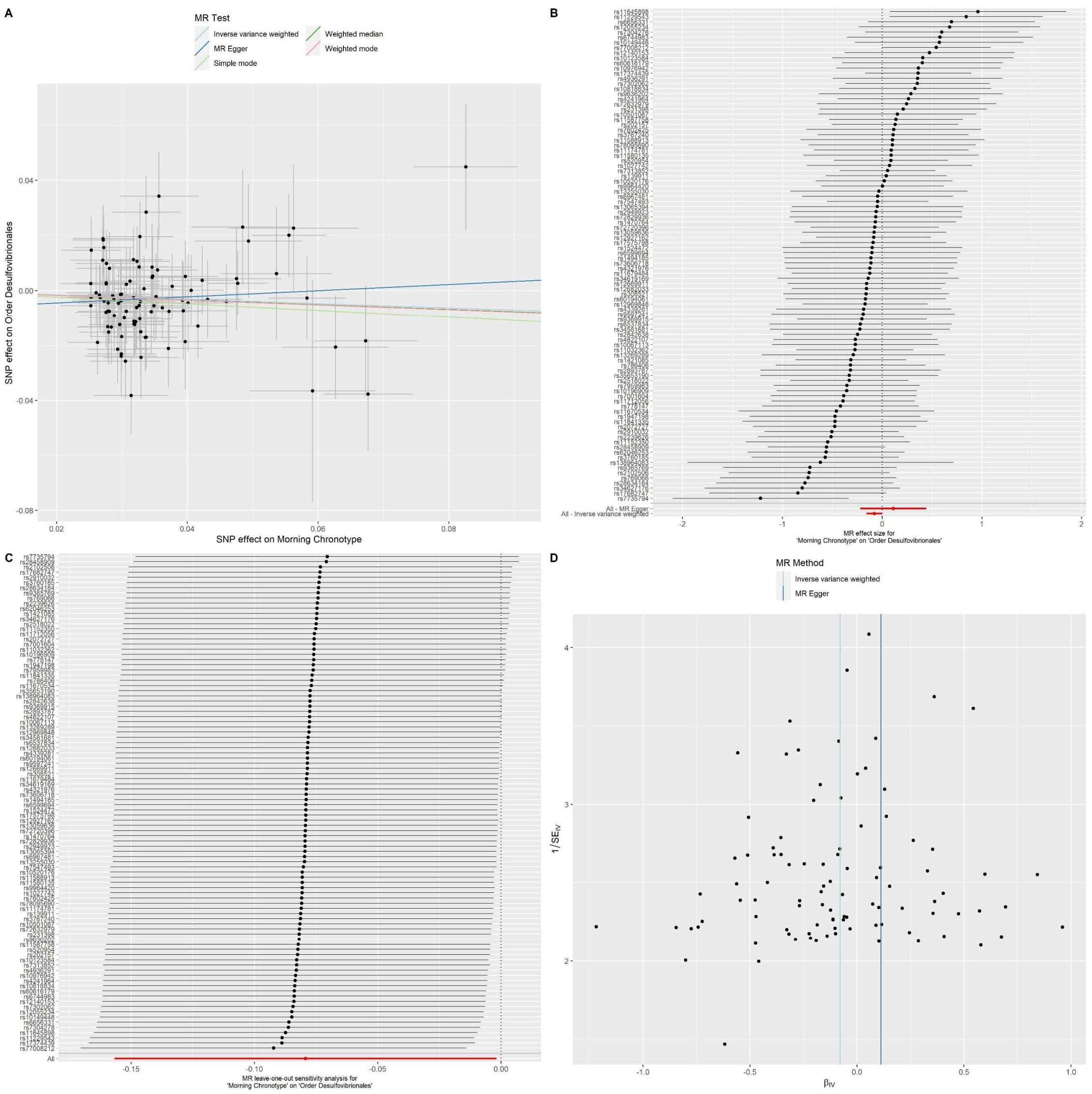

Figure S8. Mendelian randomization plots for the relationship of morning chronotype with Order *Desulfovibrionales*

Note: A, Scatterplot of SNP effects on Order *Desulfovibrionales* with the slope of each line corresponding to estimated MR effect (Inverse Variance Weighted, Weight Median, MR-Egger, Weighted Mode, and Simple Mode methods); B, Forest plot of individual and combined SNP MR-estimated effects sizes for relative Order *Desulfovibrionales*; C, The leave-one-out plot visualized how the causal estimates (point with horizontal line) for the effect of morning chronotype on Order *Desulfovibrionales* were influenced by the removal of single variant; D, Funnel plot assessing heterogeneity. Blue line represents the inverse-variance weighted estimate, and dark blue line represents the MR-Egger estimate.

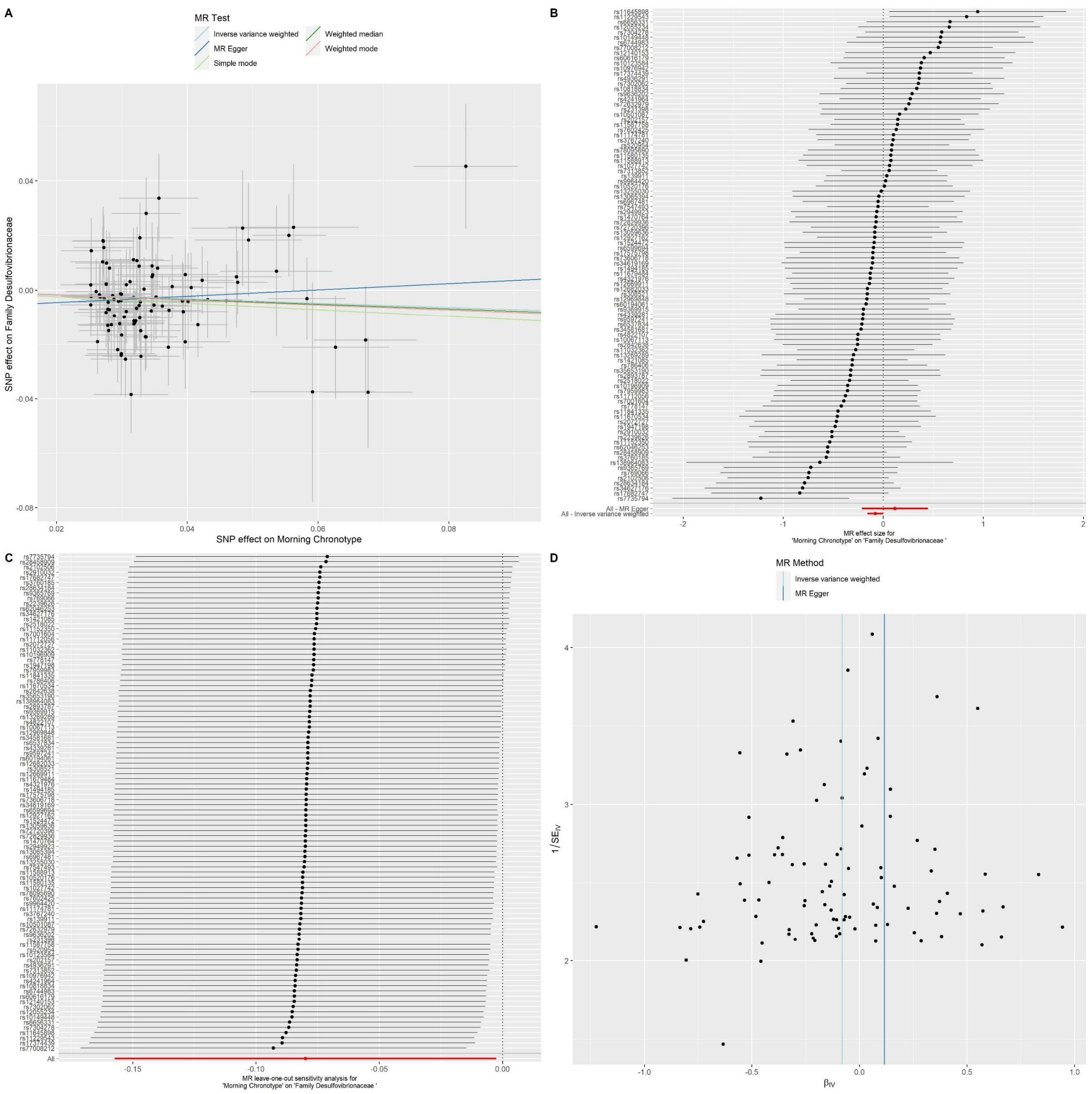

Figure S9. Mendelian randomization plots for the relationship of morning chronotype with Family *Desulfovibrionaceae*

Note: A, Scatterplot of SNP effects on Family *Desulfovibrionaceae* with the slope of each line corresponding to estimated MR effect (Inverse Variance Weighted, Weight Median, MR-Egger, Weighted Mode, and Simple Mode methods); B, Forest plot of individual and combined SNP MR-estimated effects sizes for relative Family *Desulfovibrionaceae*; C, The leave-one-out plot visualized how the causal estimates (point with horizontal line) for the effect of morning chronotype on Family *Desulfovibrionaceae* were influenced by the removal of single variant; D, Funnel plot assessing heterogeneity. Blue line represents the inverse-variance weighted estimate, and dark blue line represents the MR-Egger estimate.

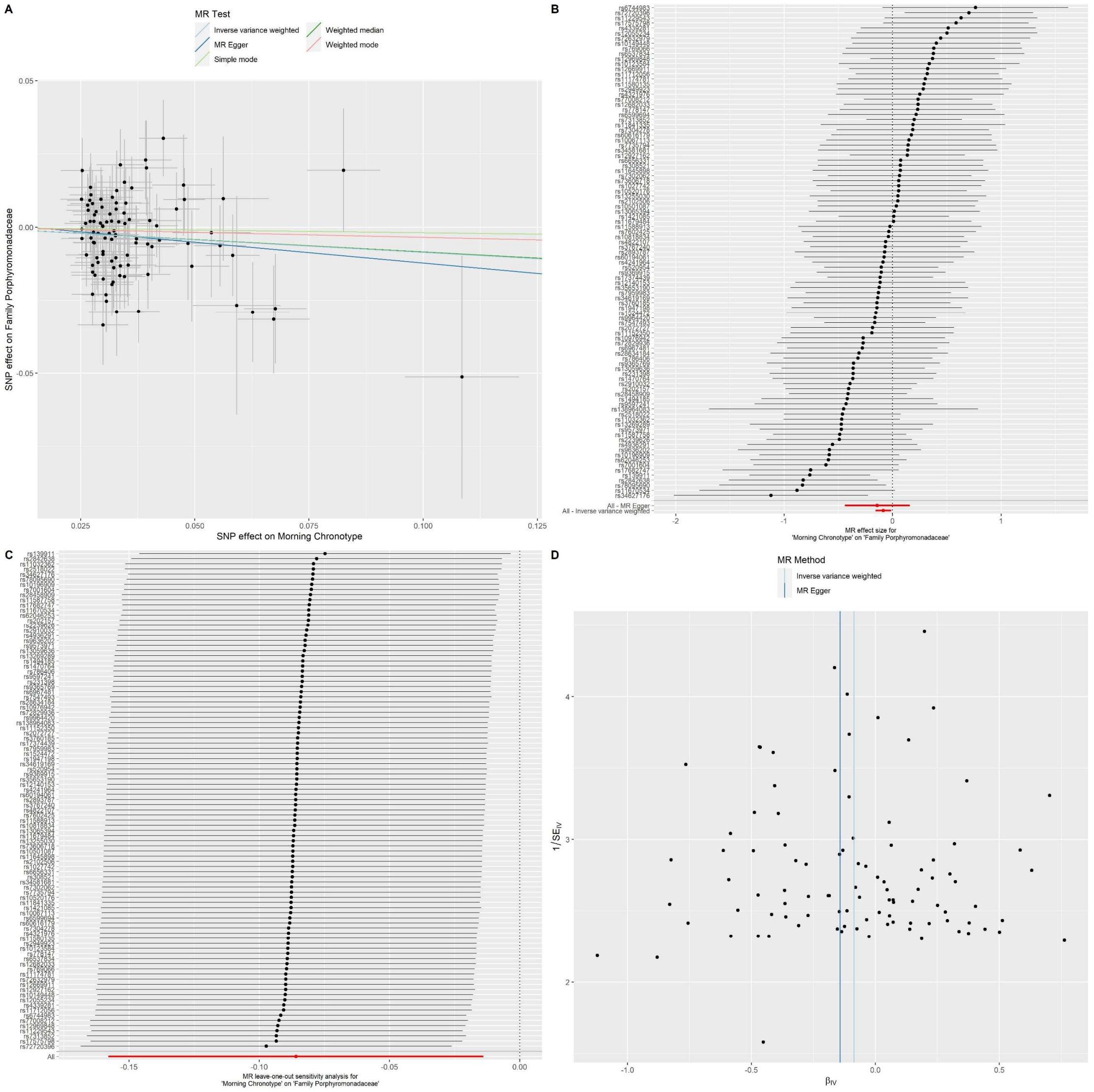

Figure S10. Mendelian randomization plots for the relationship of morning chronotype with Family *Porphyromonadaceae*

Note: A, Scatterplot of SNP effects on Family *Porphyromonadaceae* with the slope of each line corresponding to estimated MR effect (Inverse Variance Weighted, Weight Median, MR-Egger, Weighted Mode, and Simple Mode methods); B, Forest plot of individual and combined SNP MR-estimated effects sizes for relative Family *Porphyromonadaceae*; C, The leave-one-out plot visualized how the causal estimates (point with horizontal line) for the effect of morning chronotype on Family *Porphyromonadaceae* were influenced by the removal of single variant; D, Funnel plot assessing heterogeneity. Blue line represents the inverse-variance weighted estimate, and dark blue line represents the MR-Egger estimate.

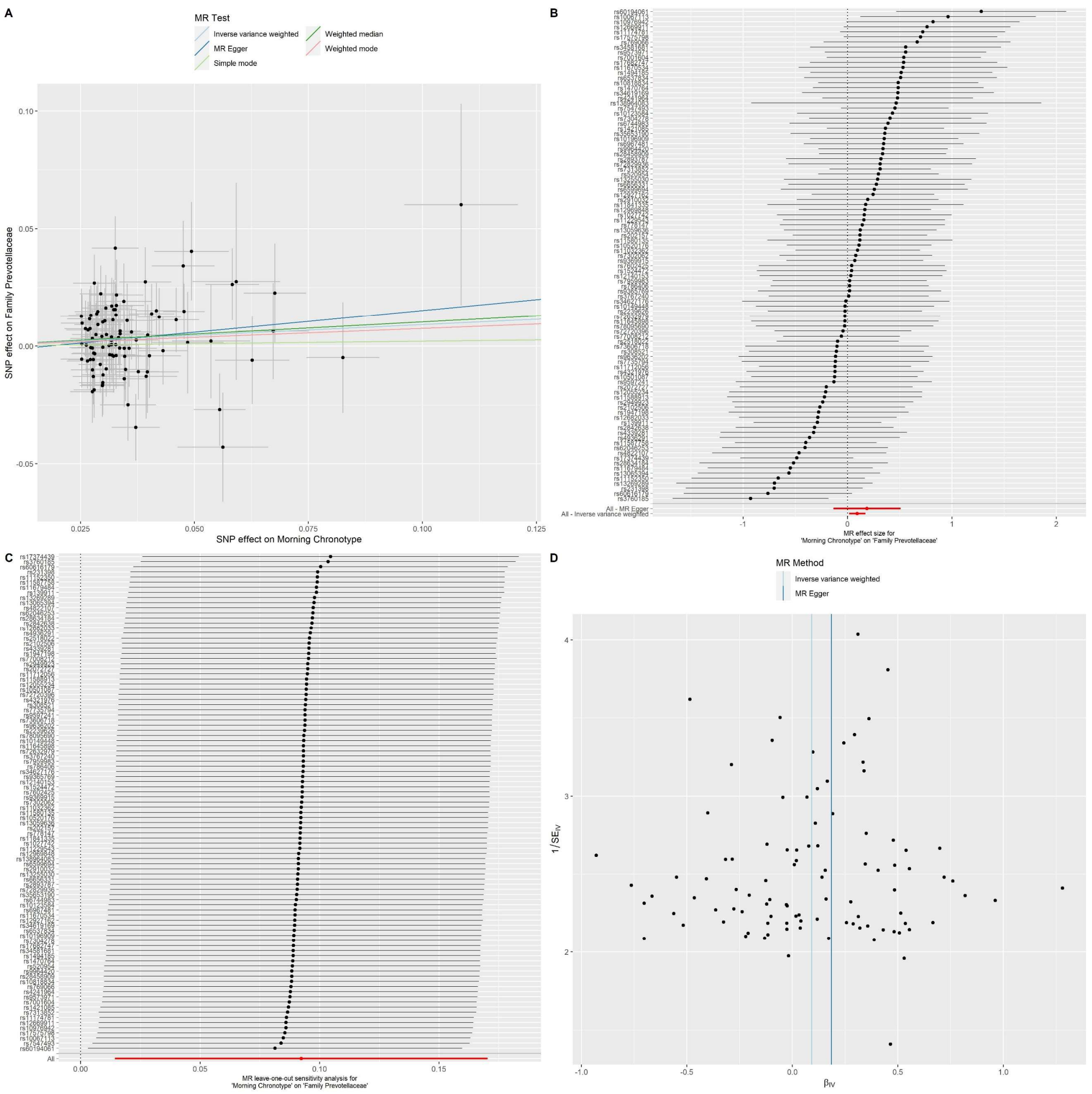

Figure S11. Mendelian randomization plots for the relationship of morning chronotype with Family *Prevotellaceae*

Note: A, Scatterplot of SNP effects on Family *Prevotellaceae* with the slope of each line corresponding to estimated MR effect (Inverse Variance Weighted, Weight Median, MR-Egger, Weighted Mode, and Simple Mode methods); B, Forest plot of individual and combined SNP MR-estimated effects sizes for relative Family *Prevotellaceae*; C, The leave-one-out plot visualized how the causal estimates (point with horizontal line) for the effect of morning chronotype on Family *Prevotellaceae* were influenced by the removal of single variant; D, Funnel plot assessing heterogeneity. Blue line represents the inverse-variance weighted estimate, and dark blue line represents the MR-Egger estimate.

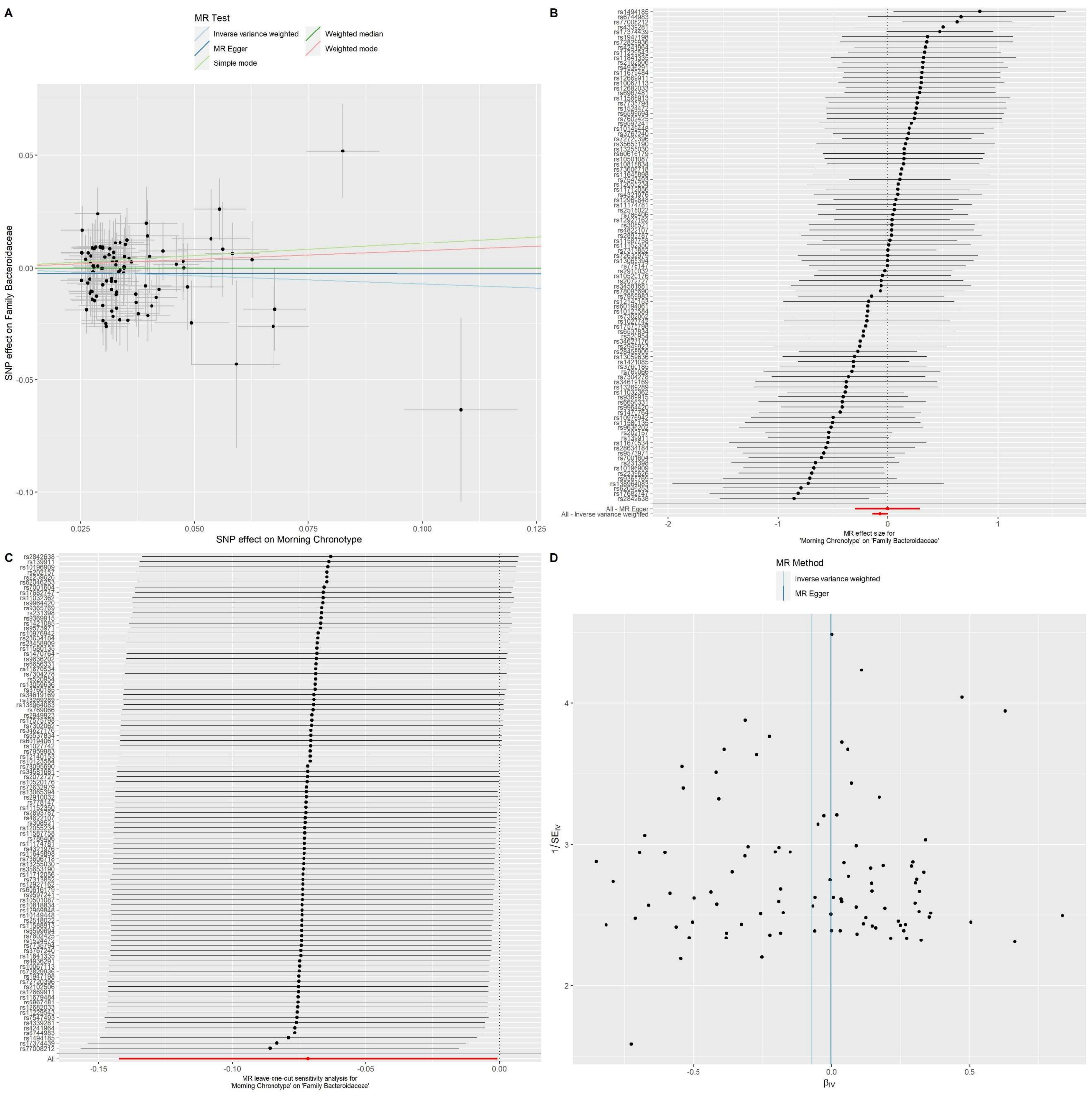

Figure S12. Mendelian randomization plots for the relationship of morning chronotype with Family *Bacteroidaceae*

Note: A, Scatterplot of SNP effects on Family *Bacteroidaceae* with the slope of each line corresponding to estimated MR effect (Inverse Variance Weighted, Weight Median, MR-Egger, Weighted Mode, and Simple Mode methods); B, Forest plot of individual and combined SNP MR-estimated effects sizes for relative Family *Bacteroidaceae*; C, The leave-one-out plot visualized how the causal estimates (point with horizontal line) for the effect of morning chronotype on Family *Bacteroidaceae* were influenced by the removal of single variant; D, Funnel plot assessing heterogeneity. Blue line represents the inverse-variance weighted estimate, and dark blue line represents the MR-Egger estimate.

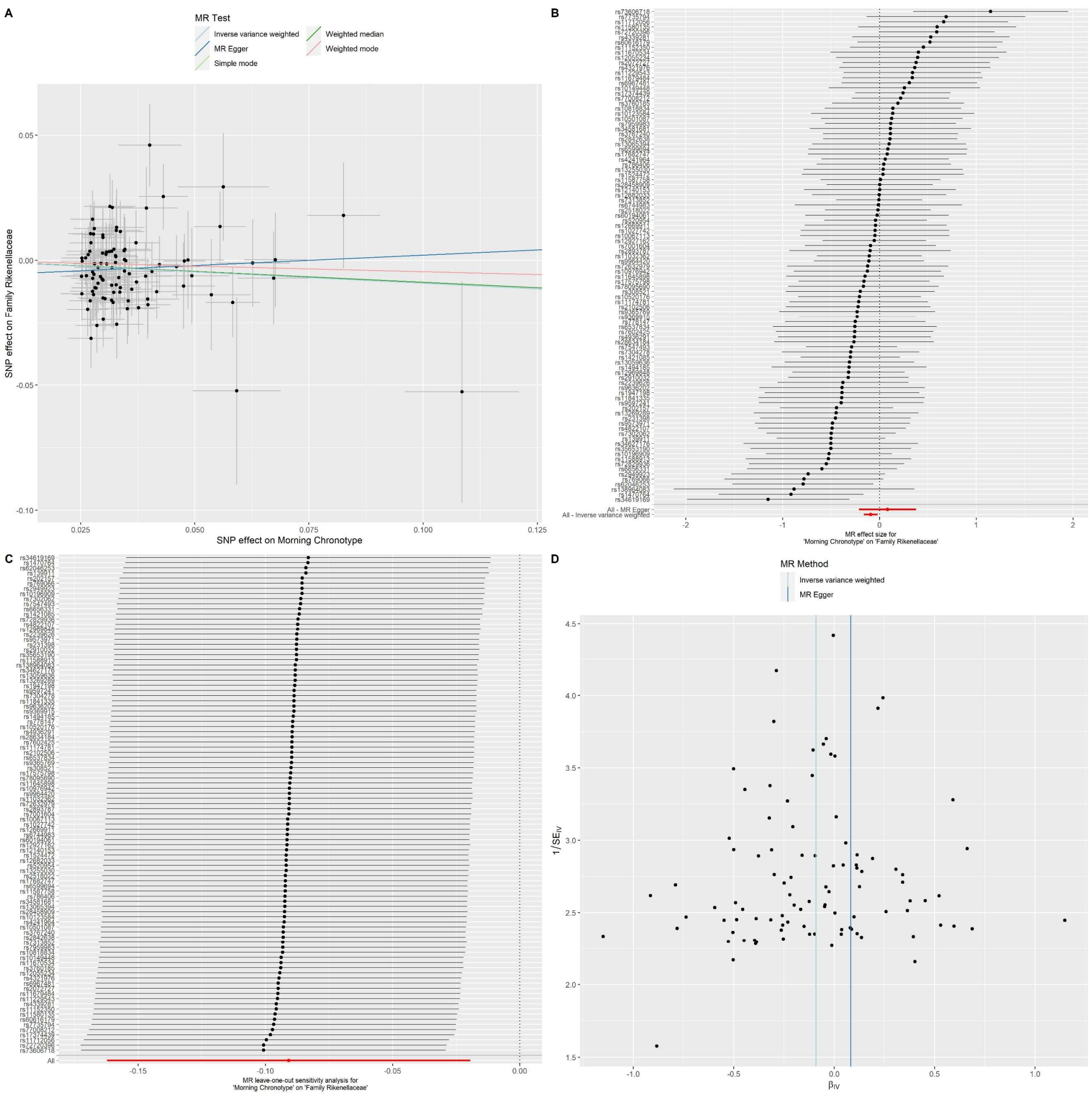

Figure S13. Mendelian randomization plots for the relationship of morning chronotype with Family *Rikenellaceae*

Note: A, Scatterplot of SNP effects on Family *Rikenellaceae* with the slope of each line corresponding to estimated MR effect (Inverse Variance Weighted, Weight Median, MR-Egger, Weighted Mode, and Simple Mode methods); B, Forest plot of individual and combined SNP MR-estimated effects sizes for relative Family *Rikenellaceae*; C, The leave-one-out plot visualized how the causal estimates (point with horizontal line) for the effect of morning chronotype on Family *Rikenellaceae* were influenced by the removal of single variant; D, Funnel plot assessing heterogeneity. Blue line represents the inverse-variance weighted estimate, and dark blue line represents the MR-Egger estimate.

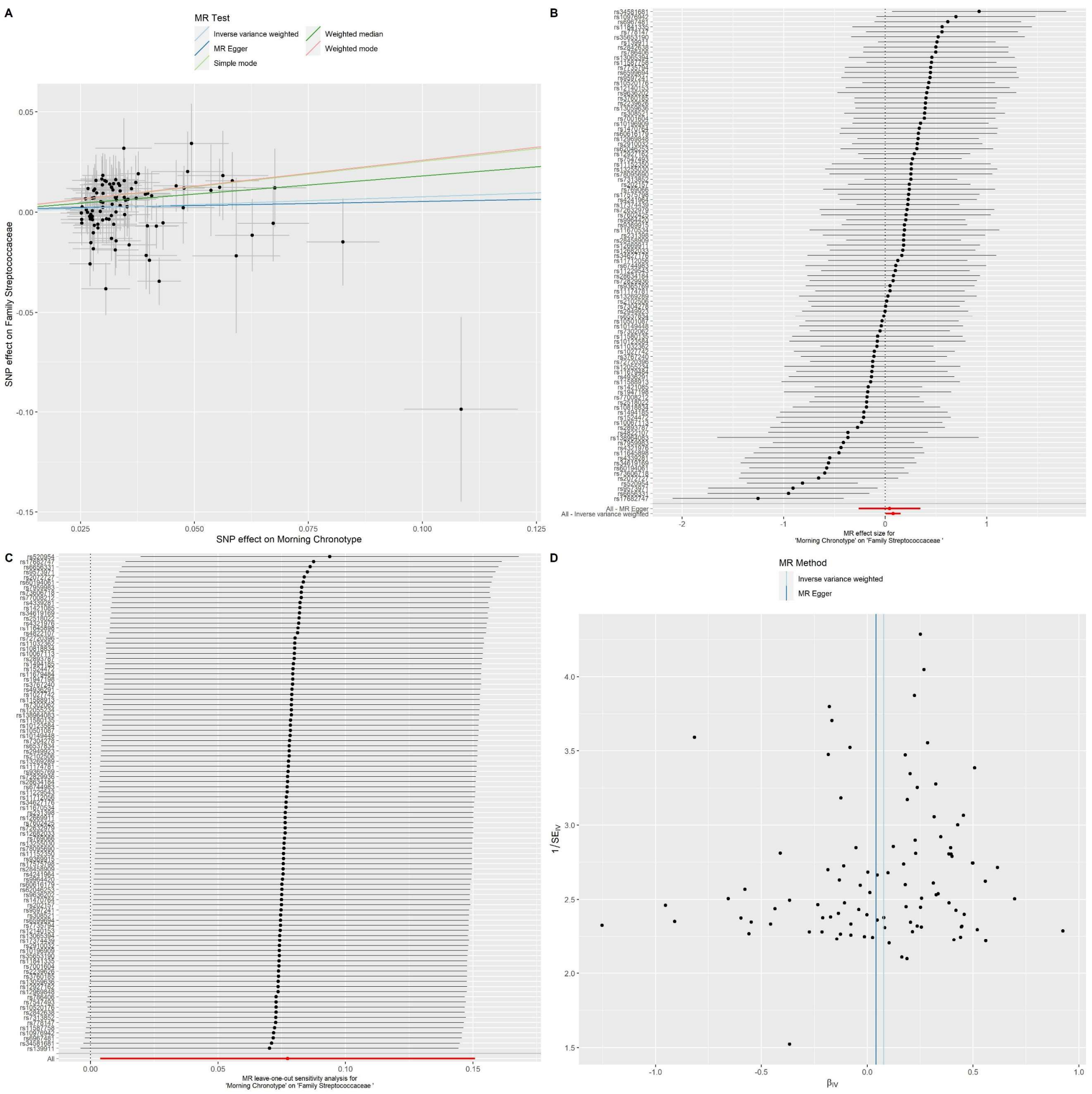

Figure S14. Mendelian randomization plots for the relationship of morning chronotype with Family *Streptococcaceae*

Note: A, Scatterplot of SNP effects on Family *Streptococcaceae* with the slope of each line corresponding to estimated MR effect (Inverse Variance Weighted, Weight Median, MR-Egger, Weighted Mode, and Simple Mode methods); B, Forest plot of individual and combined SNP MR-estimated effects sizes for relative Family *Streptococcaceae*; C, The leave-one-out plot visualized how the causal estimates (point with horizontal line) for the effect of morning chronotype on Family *Streptococcaceae* were influenced by the removal of single variant; D, Funnel plot assessing heterogeneity. Blue line represents the inverse-variance weighted estimate, and dark blue line represents the MR-Egger estimate.

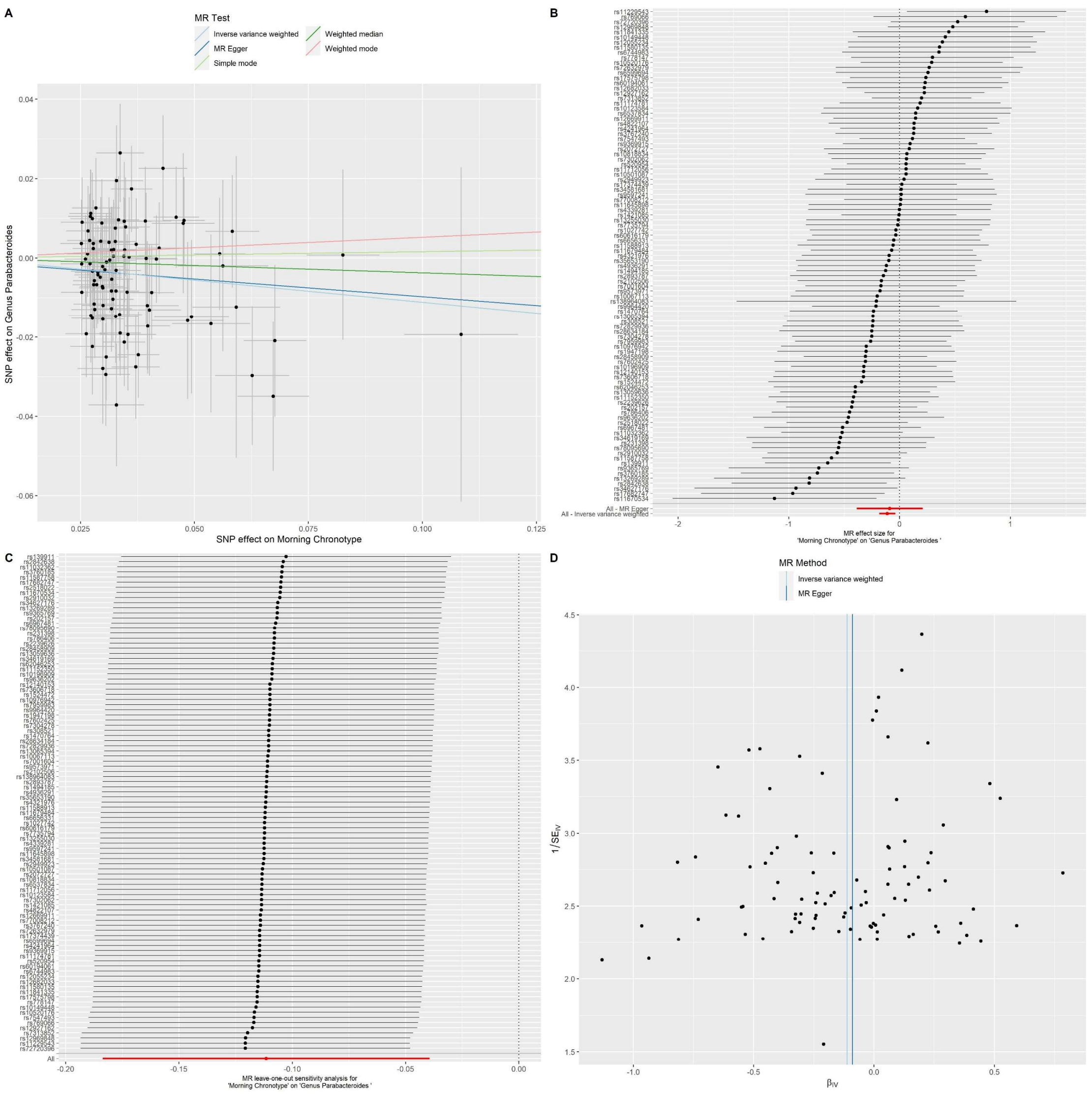

Figure S15. Mendelian randomization plots for the relationship of morning chronotype with Genus *Parabacteroides*

Note: A, Scatterplot of SNP effects on Genus *Parabacteroides* with the slope of each line corresponding to estimated MR effect (Inverse Variance Weighted, Weight Median, MR-Egger, Weighted Mode, and Simple Mode methods); B, Forest plot of individual and combined SNP MR-estimated effects sizes for relative Genus *Parabacteroides*; C, The leave-one-out plot visualized how the causal estimates (point with horizontal line) for the effect of morning chronotype on Genus *Parabacteroides* were influenced by the removal of single variant; D, Funnel plot assessing heterogeneity. Blue line represents the inverse-variance weighted estimate, and dark blue line represents the MR-Egger estimate.

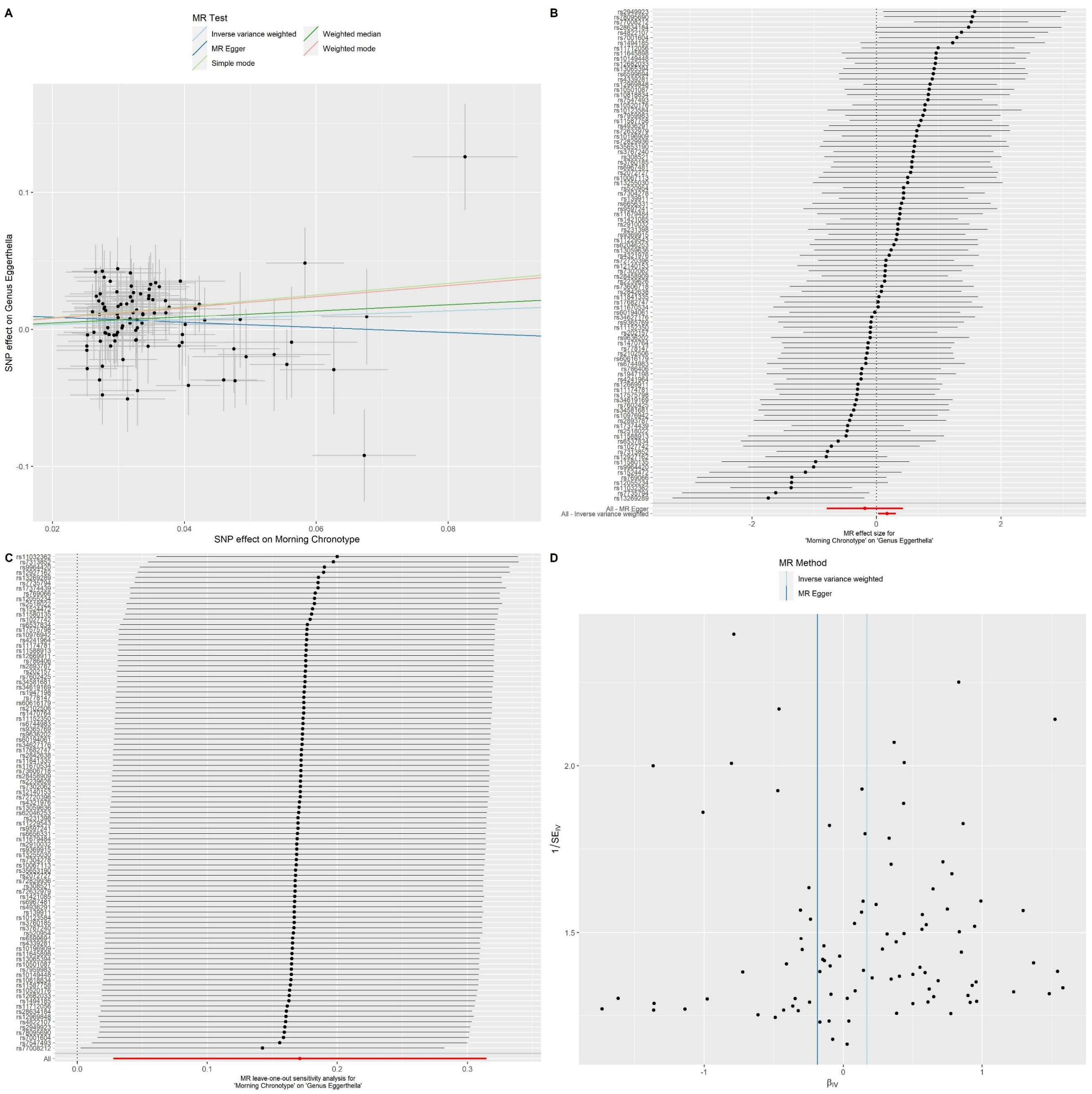

Figure S16. Mendelian randomization plots for the relationship of morning chronotype with Genus *Eggerthella*

Note: A, Scatterplot of SNP effects on Genus *Eggerthella* with the slope of each line corresponding to estimated MR effect (Inverse Variance Weighted, Weight Median, MR-Egger, Weighted Mode, and Simple Mode methods); B, Forest plot of individual and combined SNP MR-estimated effects sizes for relative Genus *Eggerthella*; C, The leave-one-out plot visualized how the causal estimates (point with horizontal line) for the effect of morning chronotype on Genus *Eggerthella* were influenced by the removal of single variant; D, Funnel plot assessing heterogeneity. Blue line represents the inverse-variance weighted estimate, and dark blue line represents the MR-Egger estimate.

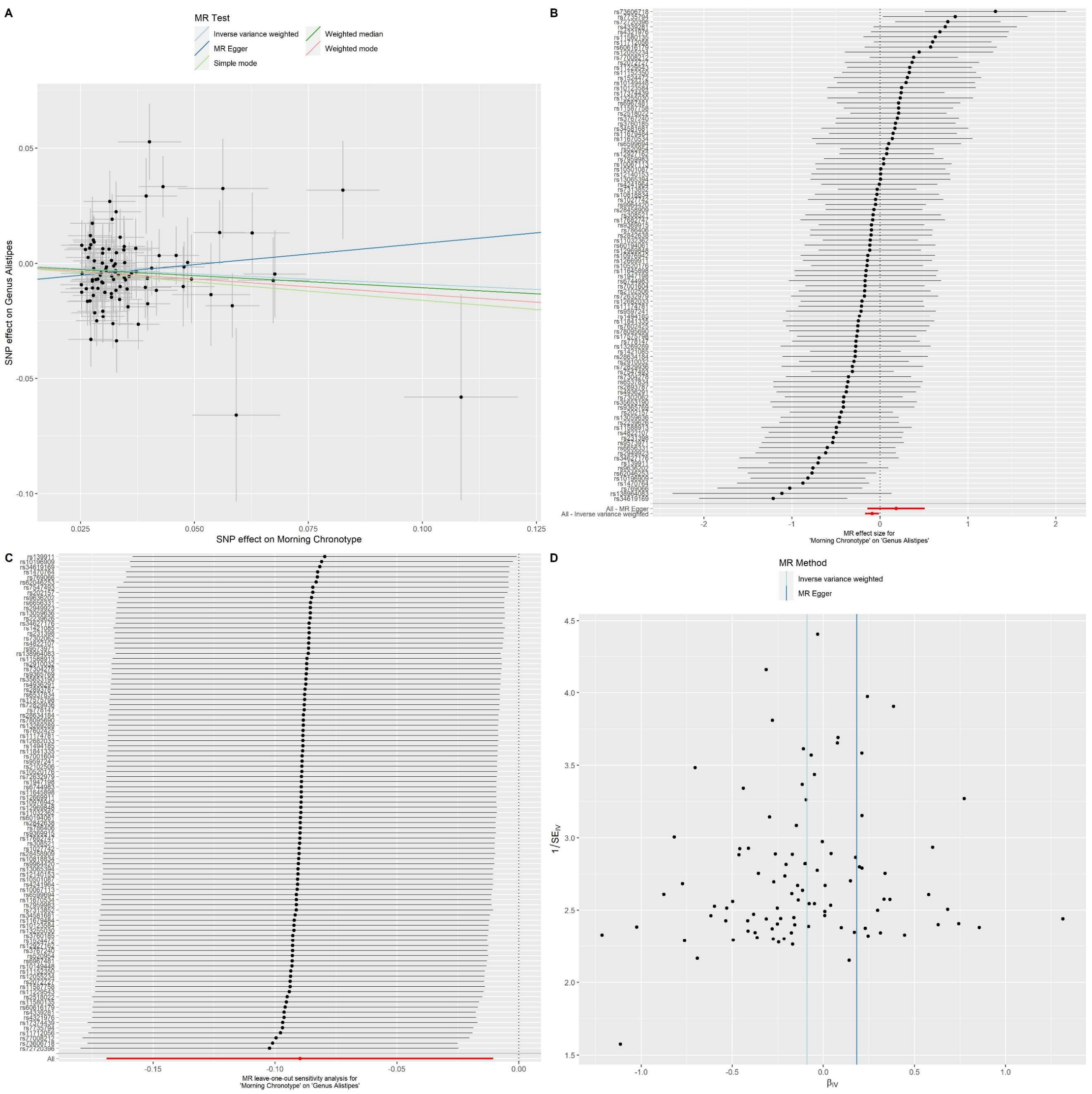

Figure S17. Mendelian randomization plots for the relationship of morning chronotype with Genus *Alistipes*

Note: A, Scatterplot of SNP effects on Genus *Alistipes* with the slope of each line corresponding to estimated MR effect (Inverse Variance Weighted, Weight Median, MR-Egger, Weighted Mode, and Simple Mode methods); B, Forest plot of individual and combined SNP MR-estimated effects sizes for relative Genus *Alistipes*; C, The leave-one-out plot visualized how the causal estimates (point with horizontal line) for the effect of morning chronotype on Genus *Alistipes* were influenced by the removal of single variant; D, Funnel plot assessing heterogeneity. Blue line represents the inverse-variance weighted estimate, and dark blue line represents the MR-Egger estimate.

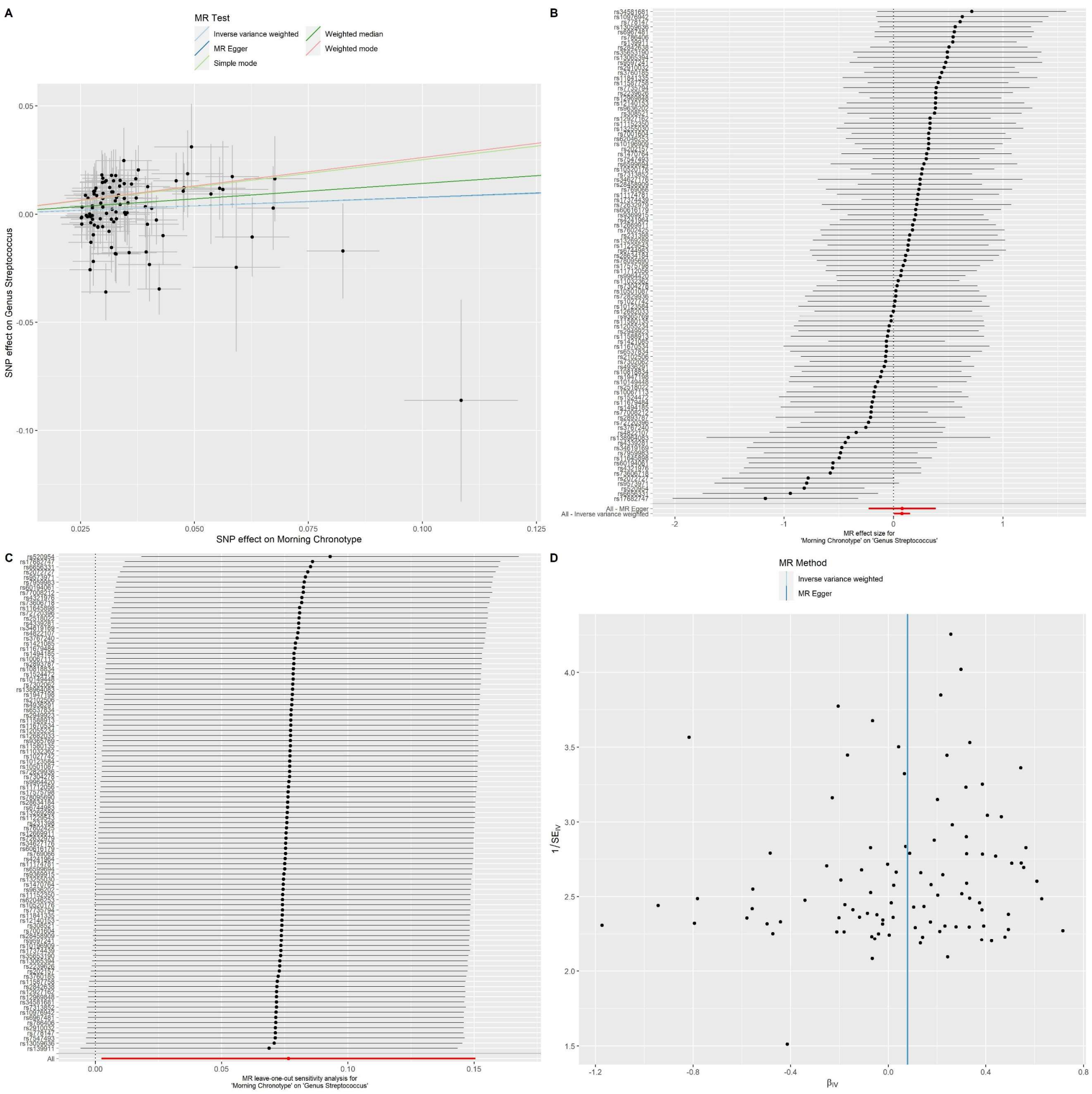

Figure S18. Mendelian randomization plots for the relationship of morning chronotype with Genus *Streptococcus*

Note: A, Scatterplot of SNP effects on Genus *Streptococcus* with the slope of each line corresponding to estimated MR effect (Inverse Variance Weighted, Weight Median, MR-Egger, Weighted Mode, and Simple Mode methods); B, Forest plot of individual and combined SNP MR-estimated effects sizes for relative Genus *Streptococcus*; C, The leave-one-out plot visualized how the causal estimates (point with horizontal line) for the effect of morning chronotype on Genus *Streptococcus* were influenced by the removal of single variant; D, Funnel plot assessing heterogeneity. Blue line represents the inverse-variance weighted estimate, and dark blue line represents the MR-Egger estimate.

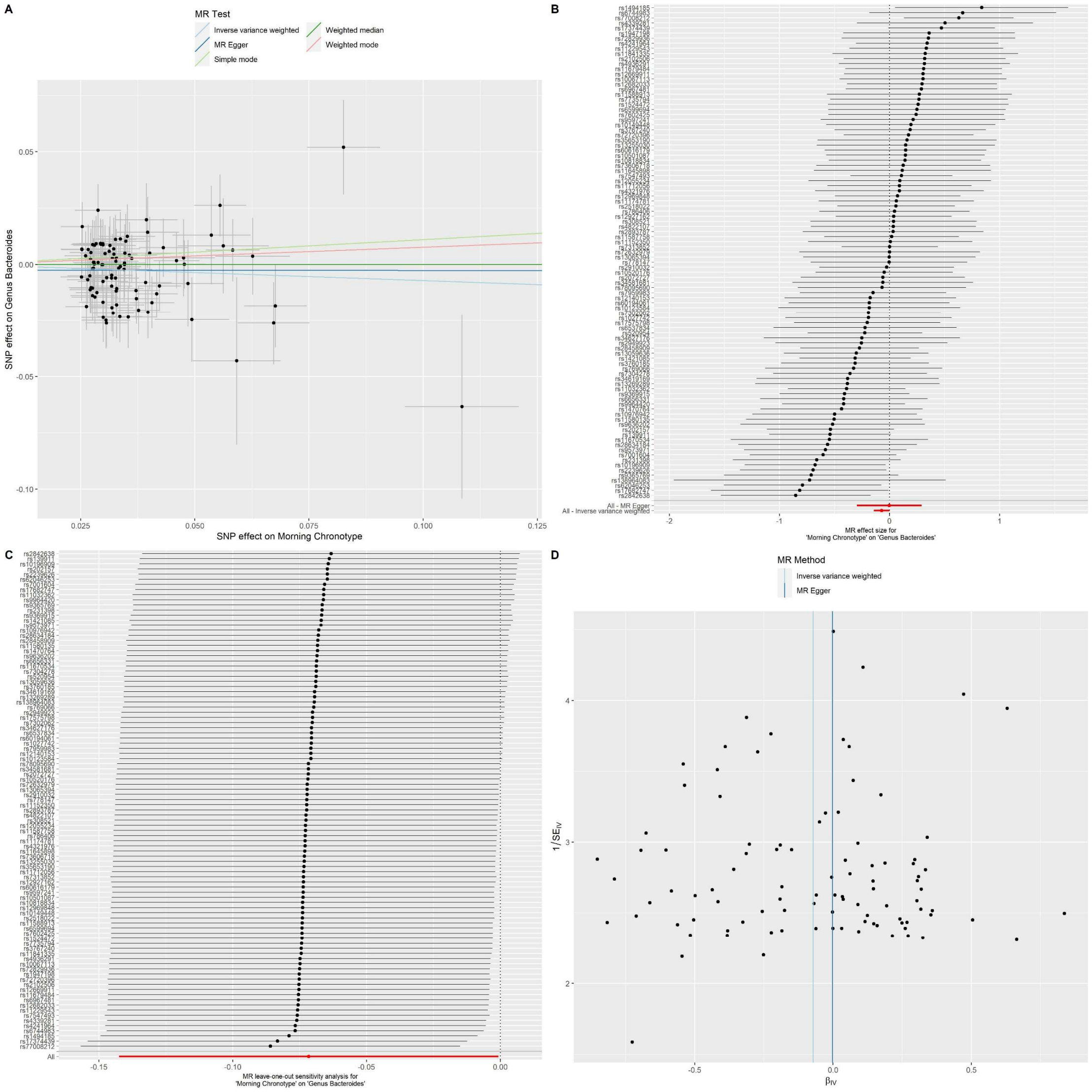

Figure S19. Mendelian randomization plots for the relationship of morning chronotype with Genus *Bacteroides*

Note: A, Scatterplot of SNP effects on Genus *Bacteroides* with the slope of each line corresponding to estimated MR effect(Inverse Variance Weighted, Weight Median, MR-Egger, Weighted Mode, and Simple Mode methods); B, Forest plot of individual and combined SNP MR-estimated effects sizes for relative Genus *Bacteroides*; C, The leave-one-out plot visualized how the causal estimates (point with horizontal line) for the effect of morning chronotype on Genus *Bacteroides* were influenced by the removal of single variant; D, Funnel plot assessing heterogeneity. Blue line represents the inverse-variance weighted estimate, and dark blue line represents the MR-Egger estimate.

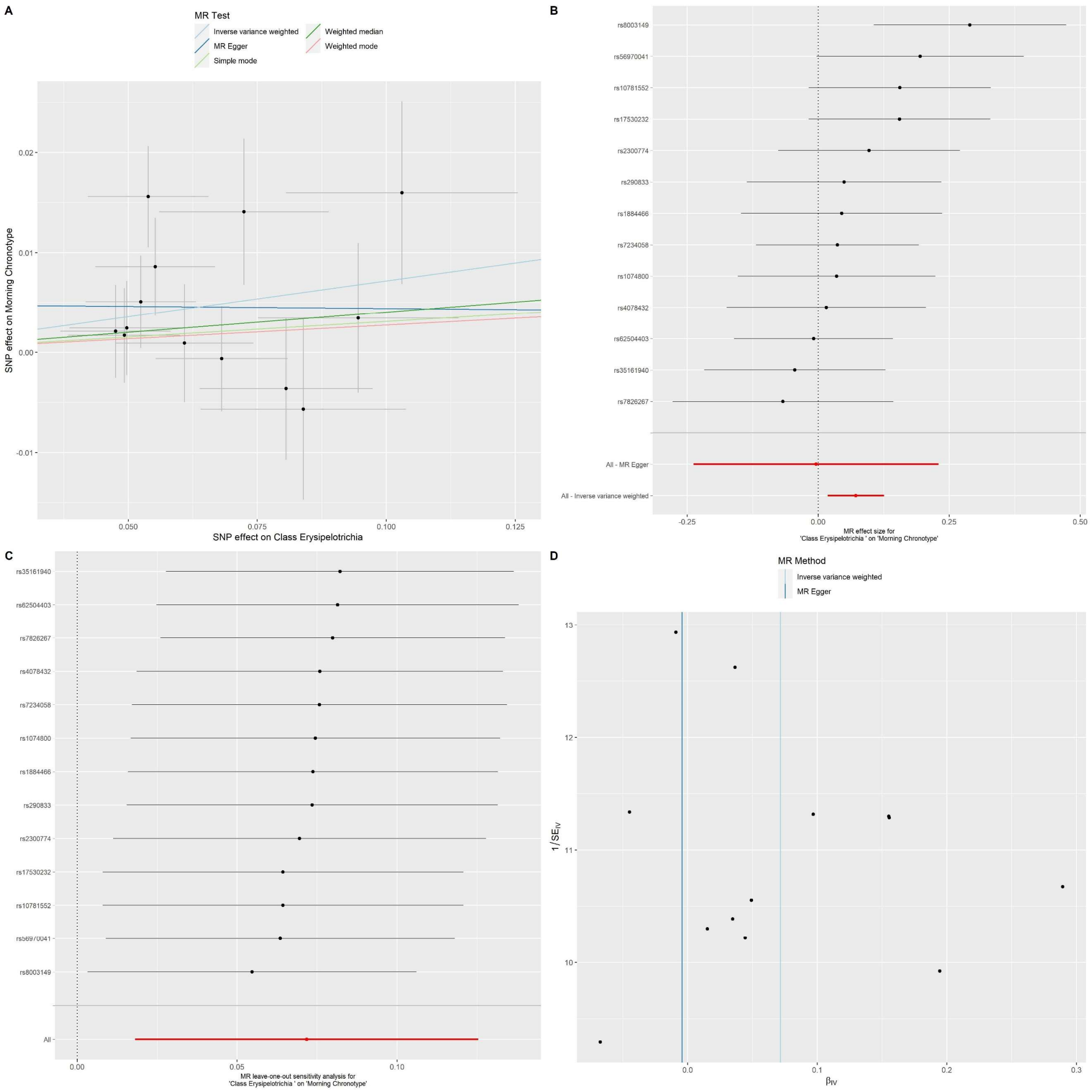

Figure S20. Mendelian randomization plots for the relationship of Class *Erysipelotrichia* with morning chronotype

Note: A, Scatterplot of SNP effects on morning chronotype with the slope of each line corresponding to estimated MR effect (Inverse Variance Weighted, Weight Median, MR-Egger, Weighted Mode, and Simple Mode methods); B, Forest plot of individual and combined SNP MR-estimated effects sizes for relative morning chronotype; C, The leave-one-out plot visualized how the causal estimates (point with horizontal line) for the effect of morning chronotype on morning chronotype were influenced by the removal of single variant; D, Funnel plot assessing heterogeneity. Blue line represents the inverse-variance weighted estimate, and dark blue line represents the MR-Egger estimate.

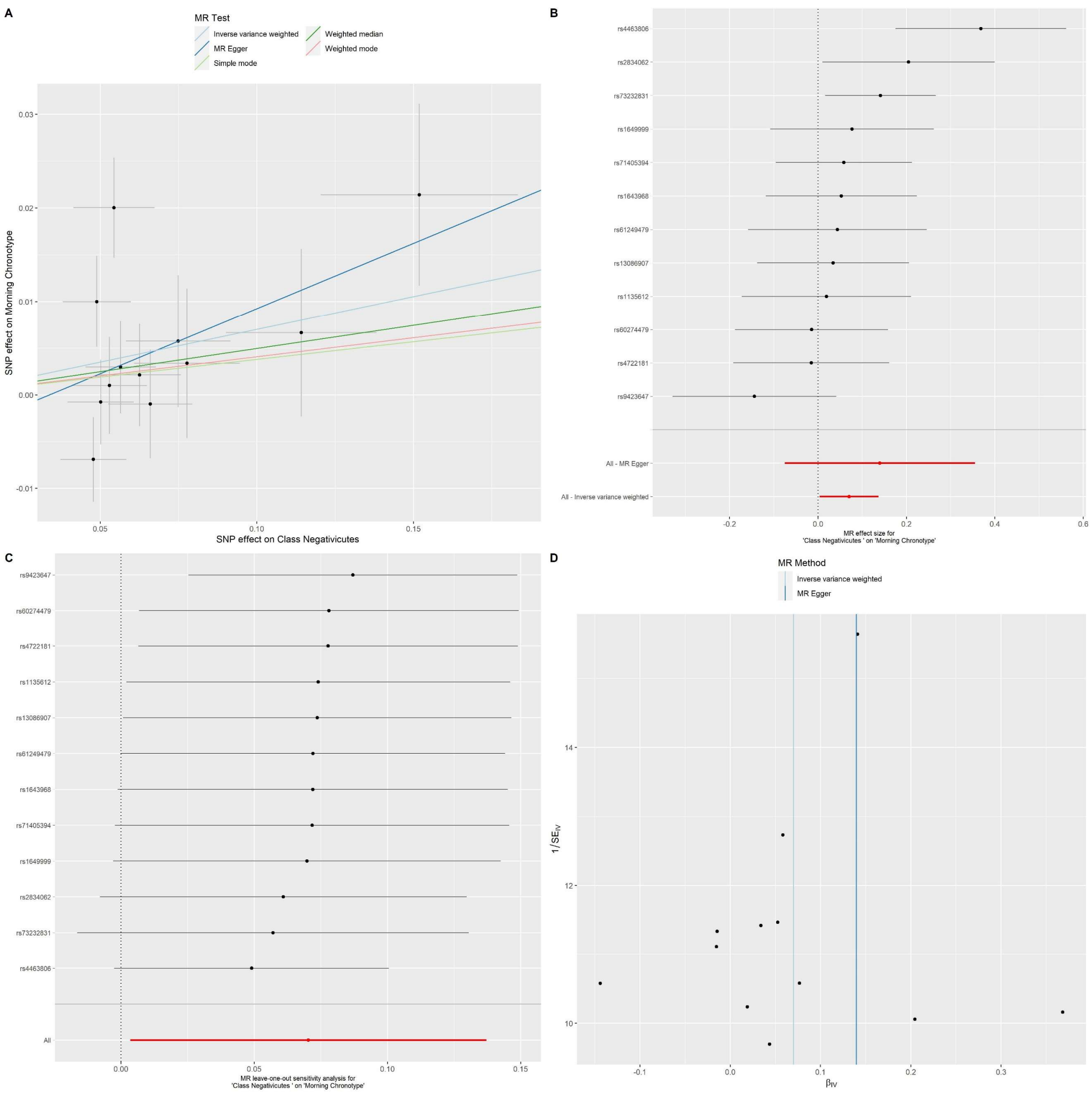

Figure S21. Mendelian randomization plots for the relationship of Class *Negativicutes* with morning chronotype

Note: A, Scatterplot of SNP effects on morning chronotype with the slope of each line corresponding to estimated MR effect (Inverse Variance Weighted, Weight Median, MR-Egger, Weighted Mode, and Simple Mode methods); B, Forest plot of individual and combined SNP MR-estimated effects sizes for relative morning chronotype; C, The leave-one-out plot visualized how the causal estimates (point with horizontal line) for the effect of morning chronotype on morning chronotype were influenced by the removal of single variant; D, Funnel plot assessing heterogeneity. Blue line represents the inverse-variance weighted estimate, and dark blue line represents the MR-Egger estimate.

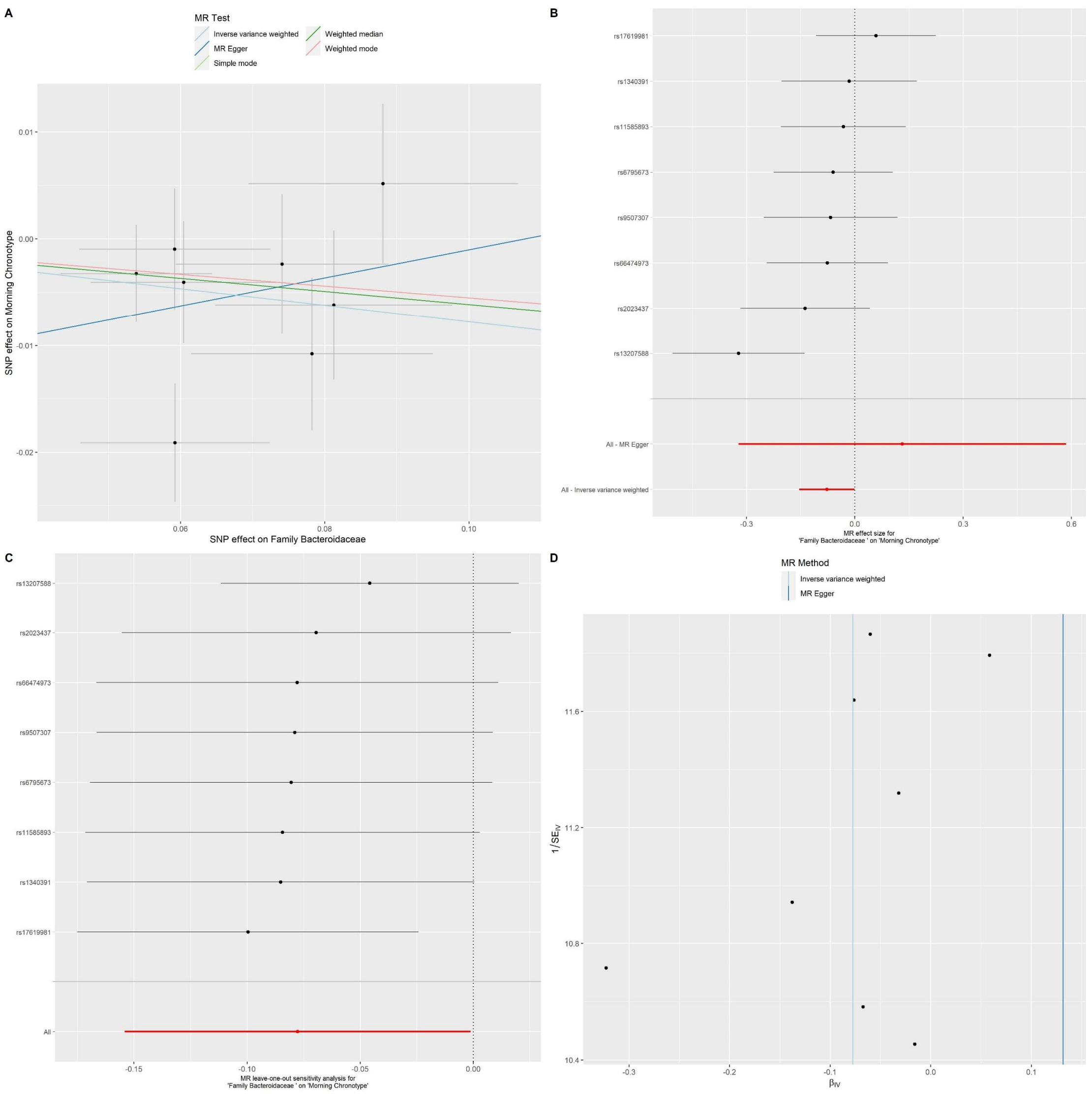

Figure S22. Mendelian randomization plots for the relationship of Family *Bacteroidaceae* with morning chronotype

Note: A, Scatterplot of SNP effects on morning chronotype with the slope of each line corresponding to estimated MR effect(Inverse Variance Weighted, Weight Median, MR-Egger, Weighted Mode, and Simple Mode methods); B, Forest plot of individual and combined SNP MR-estimated effects sizes for relative morning chronotype; C, The leave-one-out plot visualized how the causal estimates (point with horizontal line) for the effect of morning chronotype on morning chronotype were influenced by the removal of single variant; D, Funnel plot assessing heterogeneity. Blue line represents the inverse-variance weighted estimate, and dark blue line represents the MR-Egger estimate.

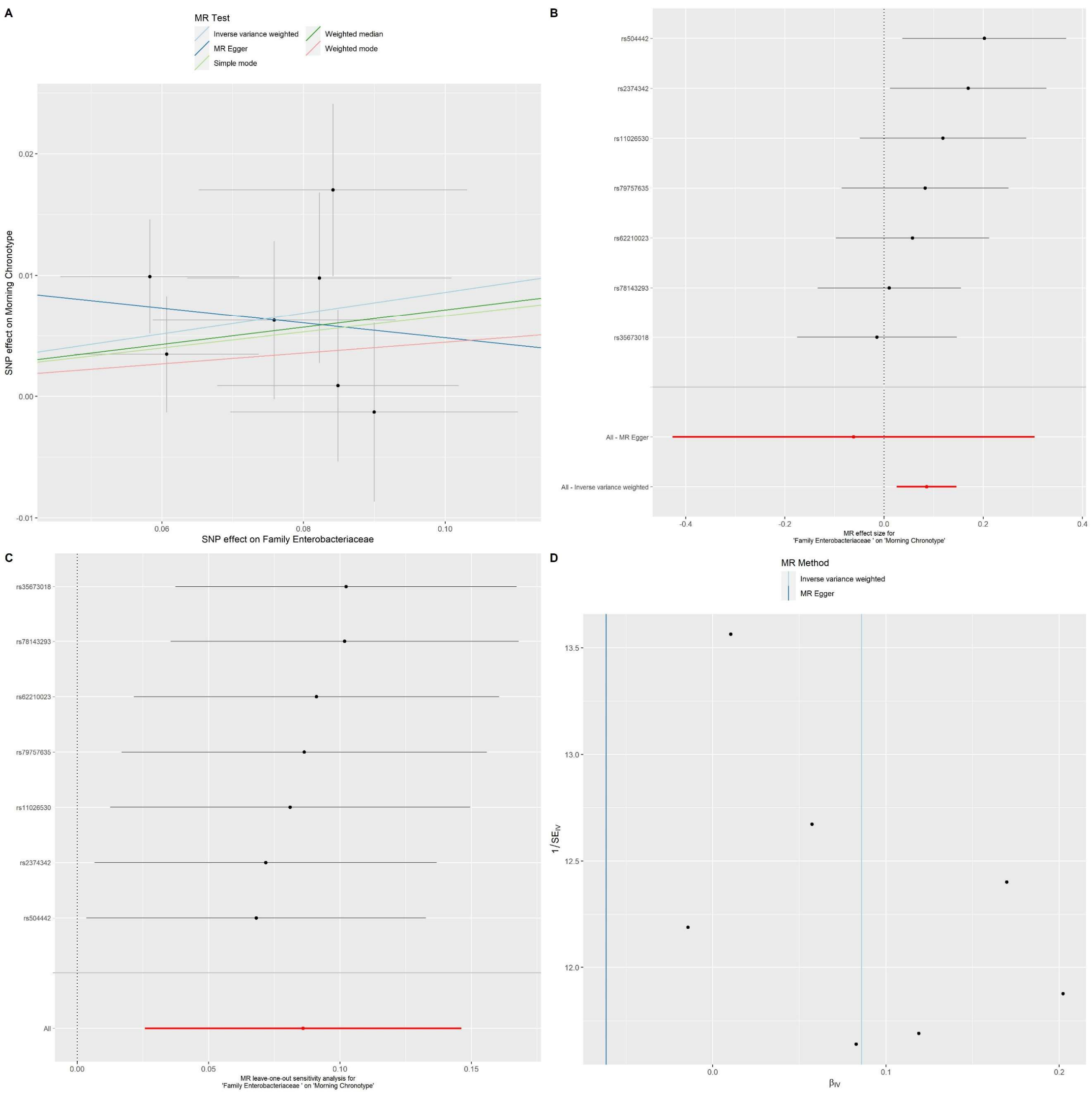

Figure S23. Mendelian randomization plots for the relationship of Family *Enterobacteriaceae* with morning chronotype

Note: A, Scatterplot of SNP effects on morning chronotype with the slope of each line corresponding to estimated MR effect(Inverse Variance Weighted, Weight Median, MR-Egger, Weighted Mode, and Simple Mode methods); B, Forest plot of individual and combined SNP MR-estimated effects sizes for relative morning chronotype; C, The leave-one-out plot visualized how the causal estimates (point with horizontal line) for the effect of morning chronotype on morning chronotype were influenced by the removal of single variant; D, Funnel plot assessing heterogeneity. Blue line represents the inverse-variance weighted estimate, and dark blue line represents the MR-Egger estimate.

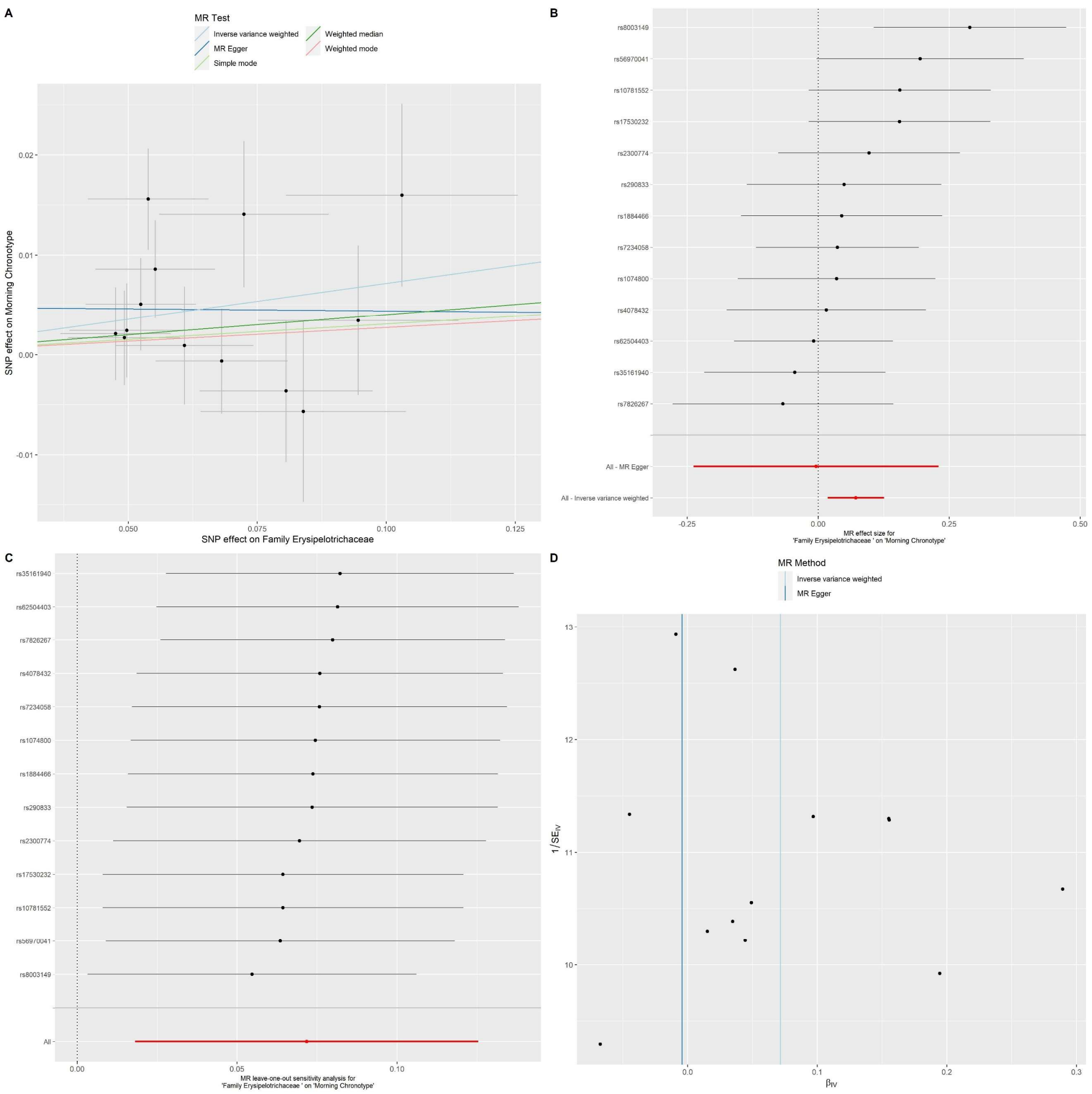

Figure S24. Mendelian randomization plots for the relationship of Family *Erysipelotrichaceae* with morning chronotype

Note: A, Scatterplot of SNP effects on morning chronotype with the slope of each line corresponding to estimated MR effect (Inverse Variance Weighted, Weight Median, MR-Egger, Weighted Mode, and Simple Mode methods); B, Forest plot of individual and combined SNP MR-estimated effects sizes for relative morning chronotype; C, The leave-one-out plot visualized how the causal estimates (point with horizontal line) for the effect of morning chronotype on morning chronotype were influenced by the removal of single variant; D, Funnel plot assessing heterogeneity. Blue line represents the inverse-variance weighted estimate, and dark blue line represents the MR-Egger estimate.

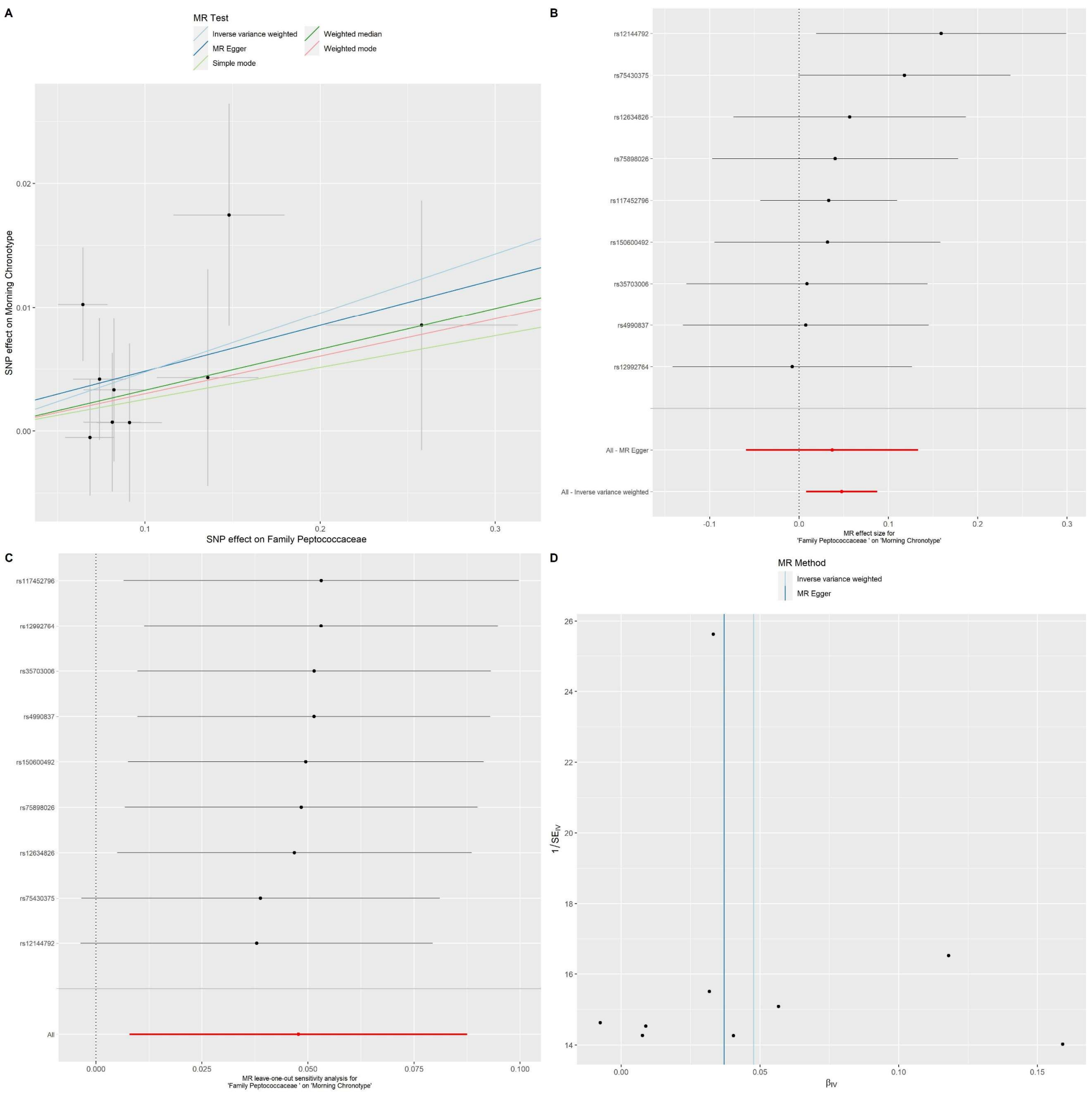

Figure S25. Mendelian randomization plots for the relationship of Family *Peptococcaceae* with morning chronotype

Note: A, Scatterplot of SNP effects on morning chronotype with the slope of each line corresponding to estimated MR effect (Inverse Variance Weighted, Weight Median, MR-Egger, Weighted Mode, and Simple Mode methods); B, Forest plot of individual and combined SNP MR-estimated effects sizes for relative morning chronotype; C, The leave-one-out plot visualized how the causal estimates (point with horizontal line) for the effect of morning chronotype on morning chronotype were influenced by the removal of single variant; D, Funnel plot assessing heterogeneity. Blue line represents the inverse-variance weighted estimate, and dark blue line represents the MR-Egger estimate.

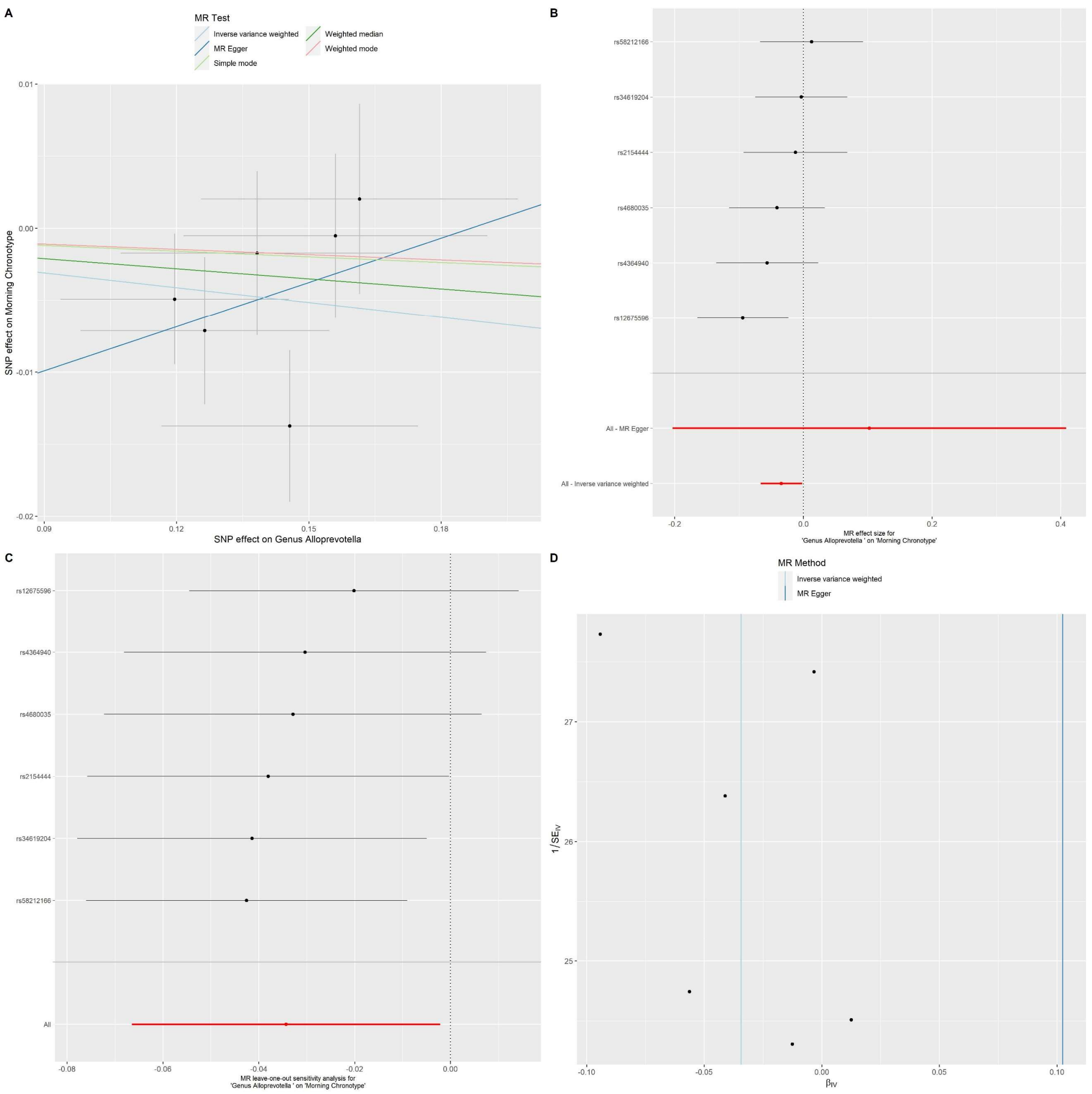

Figure S26. Mendelian randomization plots for the relationship of Genus *Alloprevotella* with morning chronotype

Note: A, Scatterplot of SNP effects on morning chronotype with the slope of each line corresponding to estimated MR effect (Inverse Variance Weighted, Weight Median, MR-Egger, Weighted Mode, and Simple Mode methods); B, Forest plot of individual and combined SNP MR-estimated effects sizes for relative morning chronotype; C, The leave-one-out plot visualized how the causal estimates (point with horizontal line) for the effect of morning chronotype on morning chronotype were influenced by the removal of single variant; D, Funnel plot assessing heterogeneity. Blue line represents the inverse-variance weighted estimate, and dark blue line represents the MR-Egger estimate.

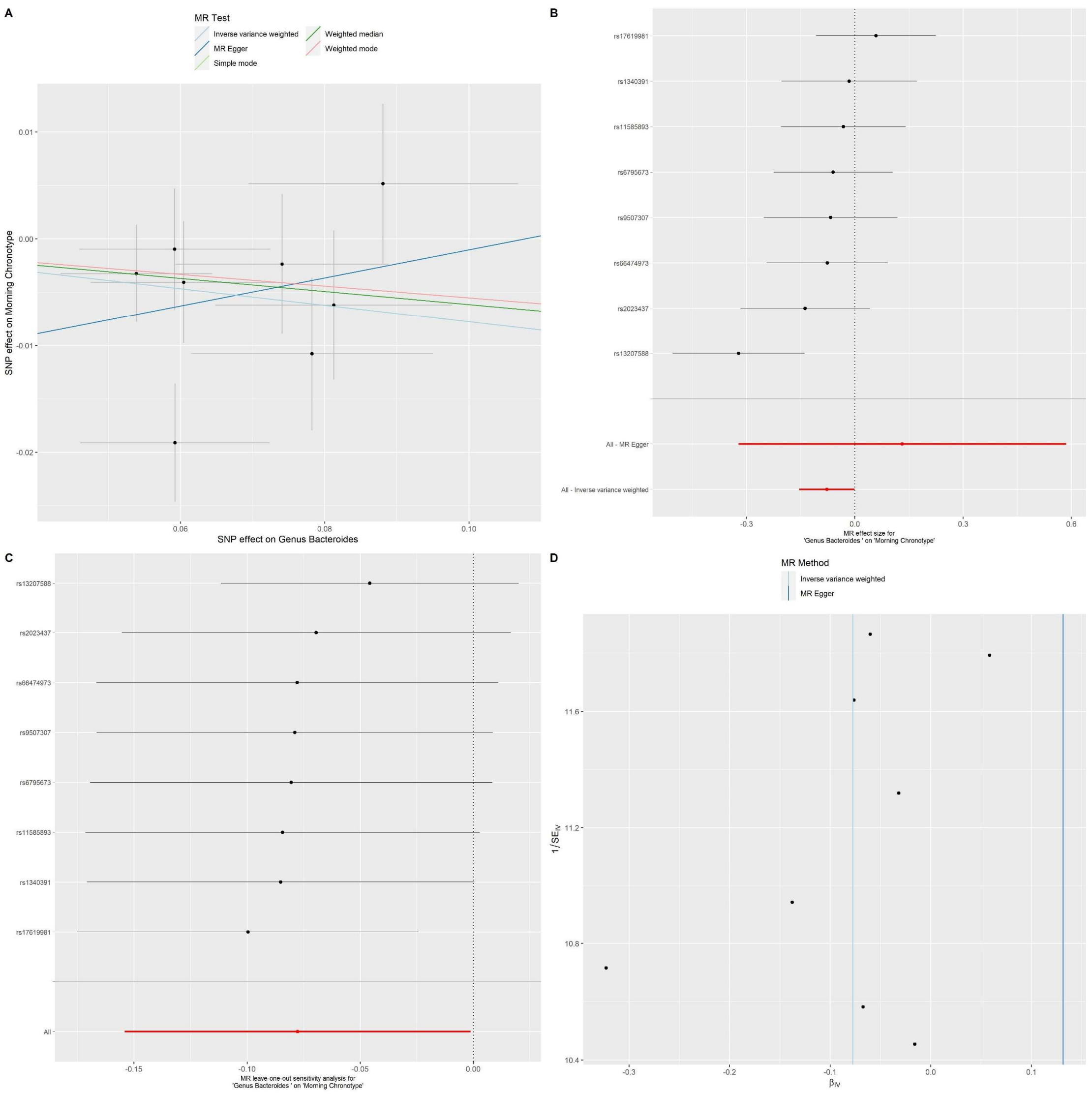

Figure S27. Mendelian randomization plots for the relationship of Genus *Bacteroides* with morning chronotype

Note: A, Scatterplot of SNP effects on morning chronotype with the slope of each line corresponding to estimated MR effect (Inverse Variance Weighted, Weight Median, MR-Egger, Weighted Mode, and Simple Mode methods); B, Forest plot of individual and combined SNP MR-estimated effects sizes for relative morning chronotype; C, The leave-one-out plot visualized how the causal estimates (point with horizontal line) for the effect of morning chronotype on morning chronotype were influenced by the removal of single variant; D, Funnel plot assessing heterogeneity. Blue line represents the inverse-variance weighted estimate, and dark blue line represents the MR-Egger estimate.

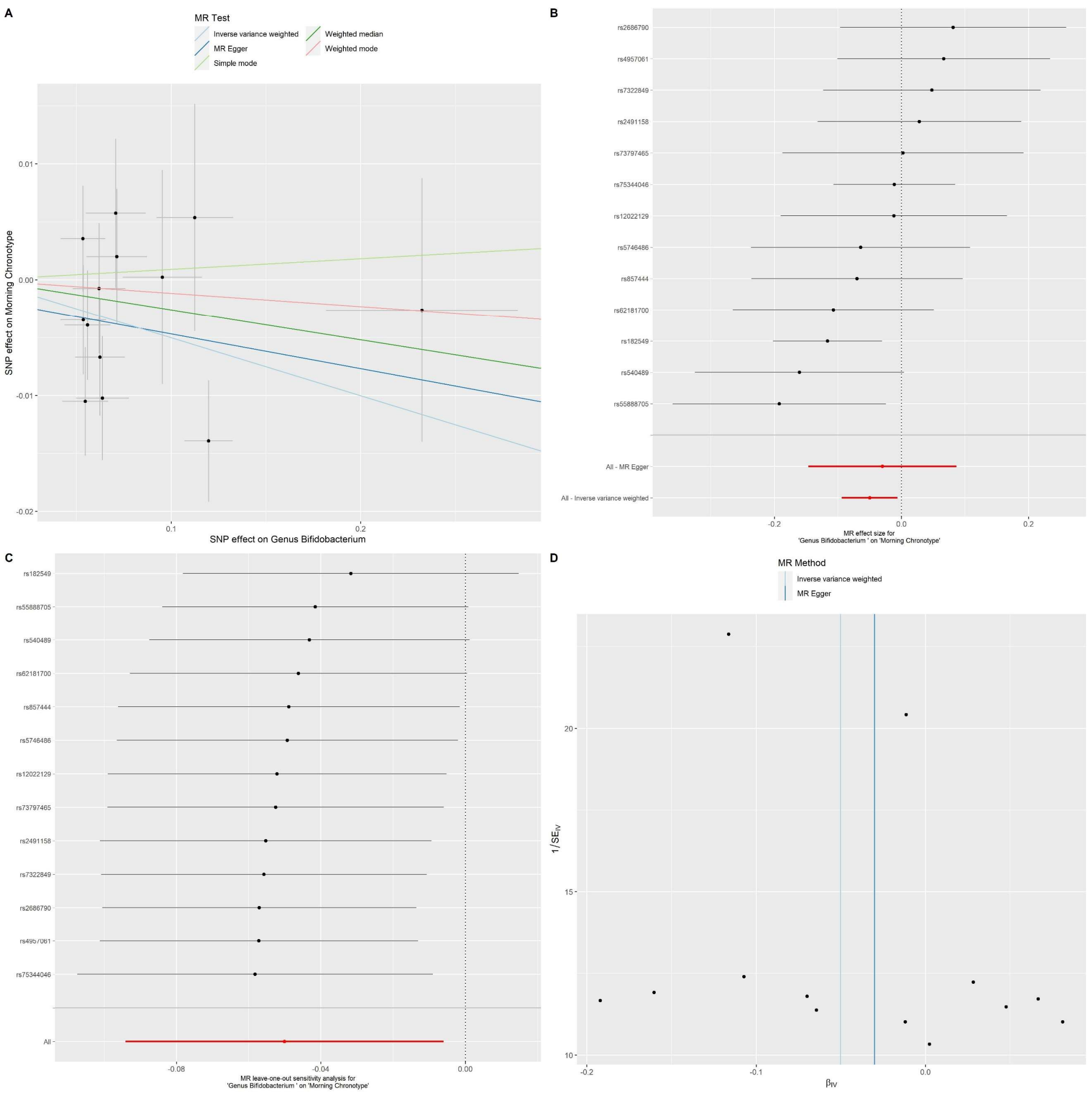

Figure S28. Mendelian randomization plots for the relationship of Genus *Bifidobacterium* with morning chronotype

Note: A, Scatterplot of SNP effects on morning chronotype with the slope of each line corresponding to estimated MR effect (Inverse Variance Weighted, Weight Median, MR-Egger, Weighted Mode, and Simple Mode methods); B, Forest plot of individual and combined SNP MR-estimated effects sizes for relative morning chronotype; C, The leave-one-out plot visualized how the causal estimates (point with horizontal line) for the effect of morning chronotype on morning chronotype were influenced by the removal of single variant; D, Funnel plot assessing heterogeneity. Blue line represents the inverse-variance weighted estimate, and dark blue line represents the MR-Egger estimate.

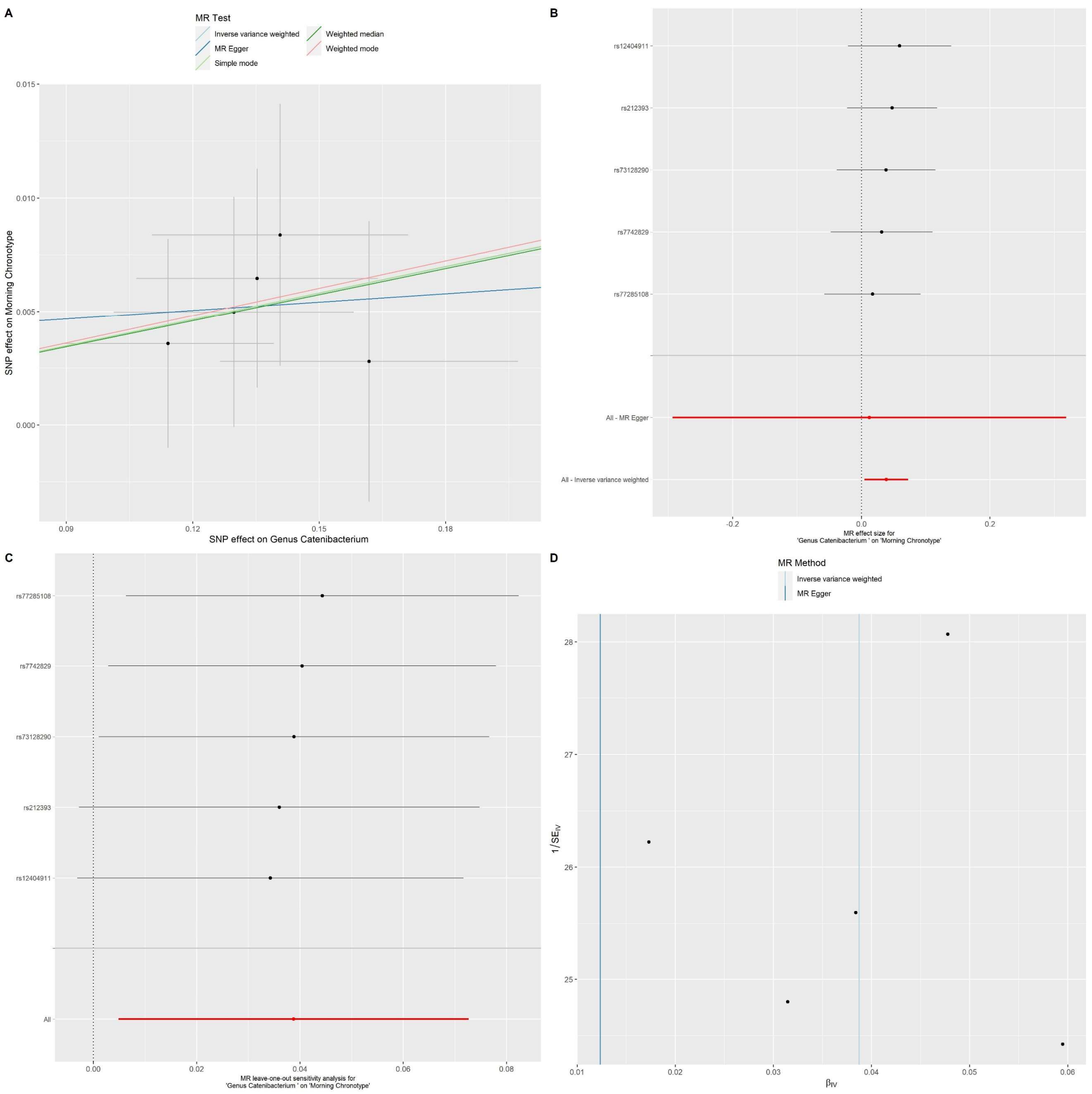

Figure S29. Mendelian randomization plots for the relationship of Genus *Catenibacterium* with morning chronotype

Note: A, Scatterplot of SNP effects on morning chronotype with the slope of each line corresponding to estimated MR effect (Inverse Variance Weighted, Weight Median, MR-Egger, Weighted Mode, and Simple Mode methods); B, Forest plot of individual and combined SNP MR-estimated effects sizes for relative morning chronotype; C, The leave-one-out plot visualized how the causal estimates (point with horizontal line) for the effect of morning chronotype on morning chronotype were influenced by the removal of single variant; D, Funnel plot assessing heterogeneity. Blue line represents the inverse-variance weighted estimate, and dark blue line represents the MR-Egger estimate.

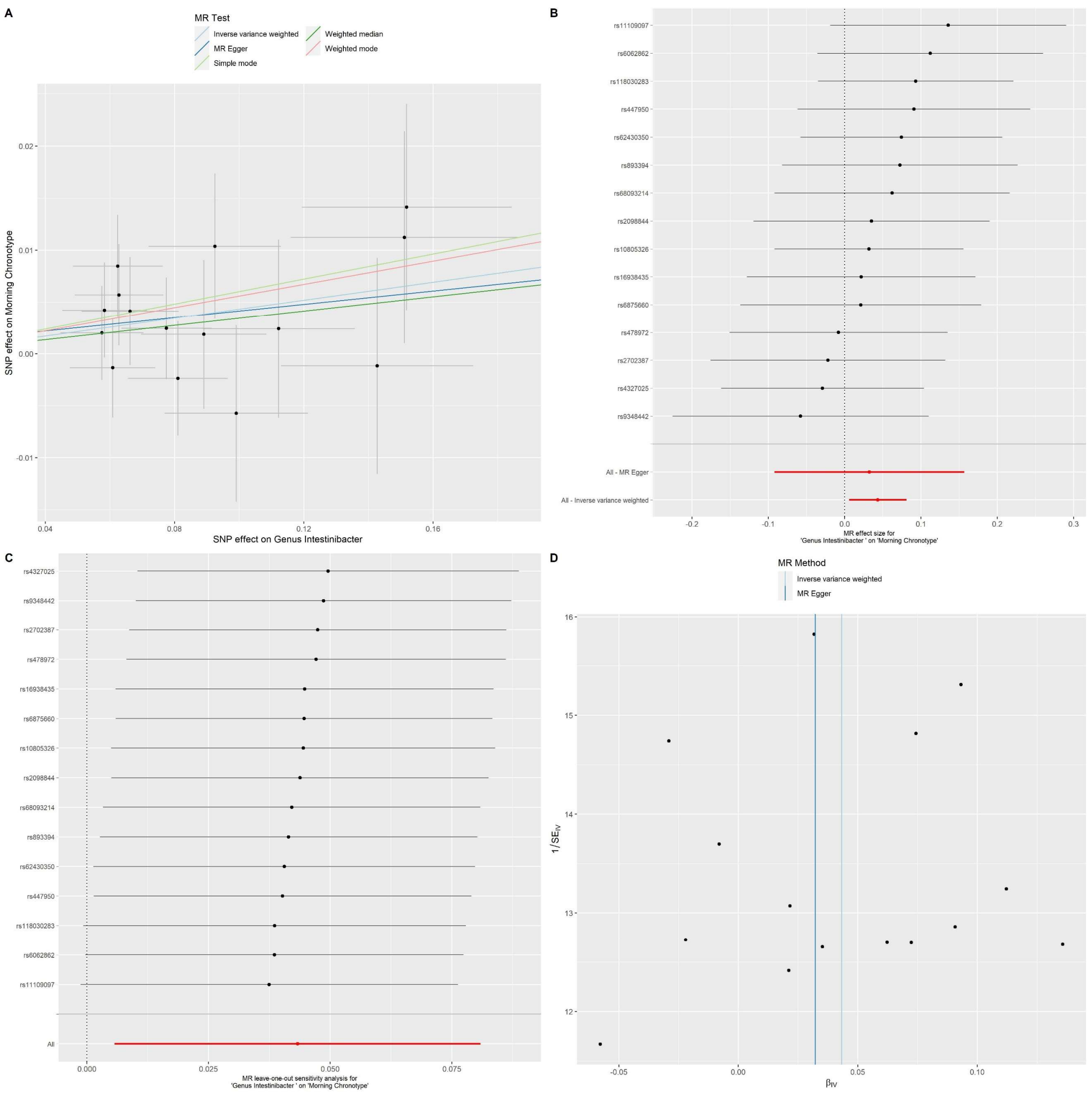

Figure S30. Mendelian randomization plots for the relationship of Genus *Intestinibacter* with morning chronotype

Note: A, Scatterplot of SNP effects on morning chronotype with the slope of each line corresponding to estimated MR effect (Inverse Variance Weighted, Weight Median, MR-Egger, Weighted Mode, and Simple Mode methods); B, Forest plot of individual and combined SNP MR-estimated effects sizes for relative morning chronotype; C, The leave-one-out plot visualized how the causal estimates (point with horizontal line) for the effect of morning chronotype on morning chronotype were influenced by the removal of single variant; D, Funnel plot assessing heterogeneity. Blue line represents the inverse-variance weighted estimate, and dark blue line represents the MR-Egger estimate.

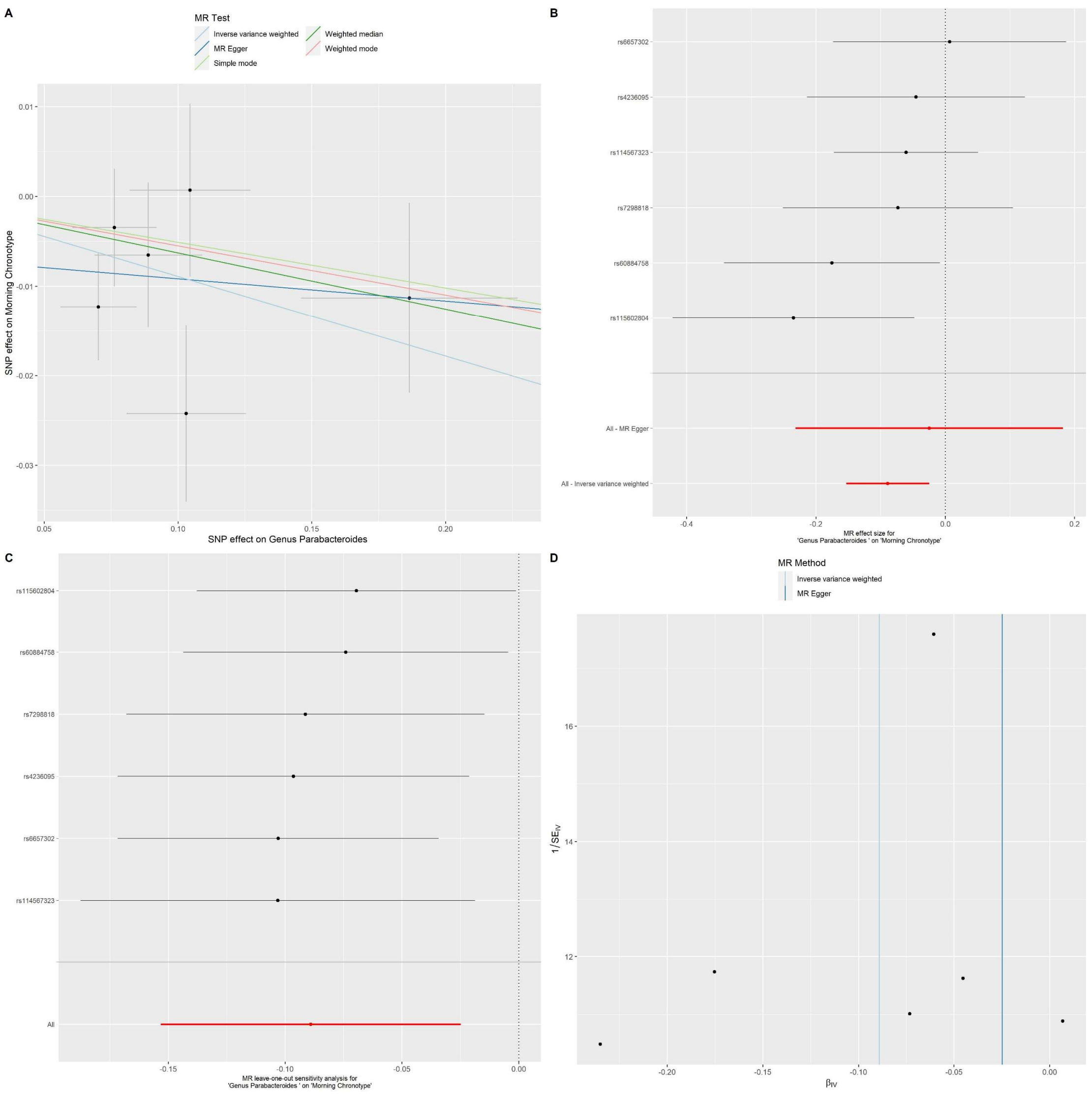

Figure S31. Mendelian randomization plots for the relationship of Genus *Parabacteroides* with morning chronotype

Note: A, Scatterplot of SNP effects on morning chronotype with the slope of each line corresponding to estimated MR effect (Inverse Variance Weighted, Weight Median, MR-Egger, Weighted Mode, and Simple Mode methods); B, Forest plot of individual and combined SNP MR-estimated effects sizes for relative morning chronotype; C, The leave-one-out plot visualized how the causal estimates (point with horizontal line) for the effect of morning chronotype on morning chronotype were influenced by the removal of single variant; D, Funnel plot assessing heterogeneity. Blue line represents the inverse-variance weighted estimate, and dark blue line represents the MR-Egger estimate.

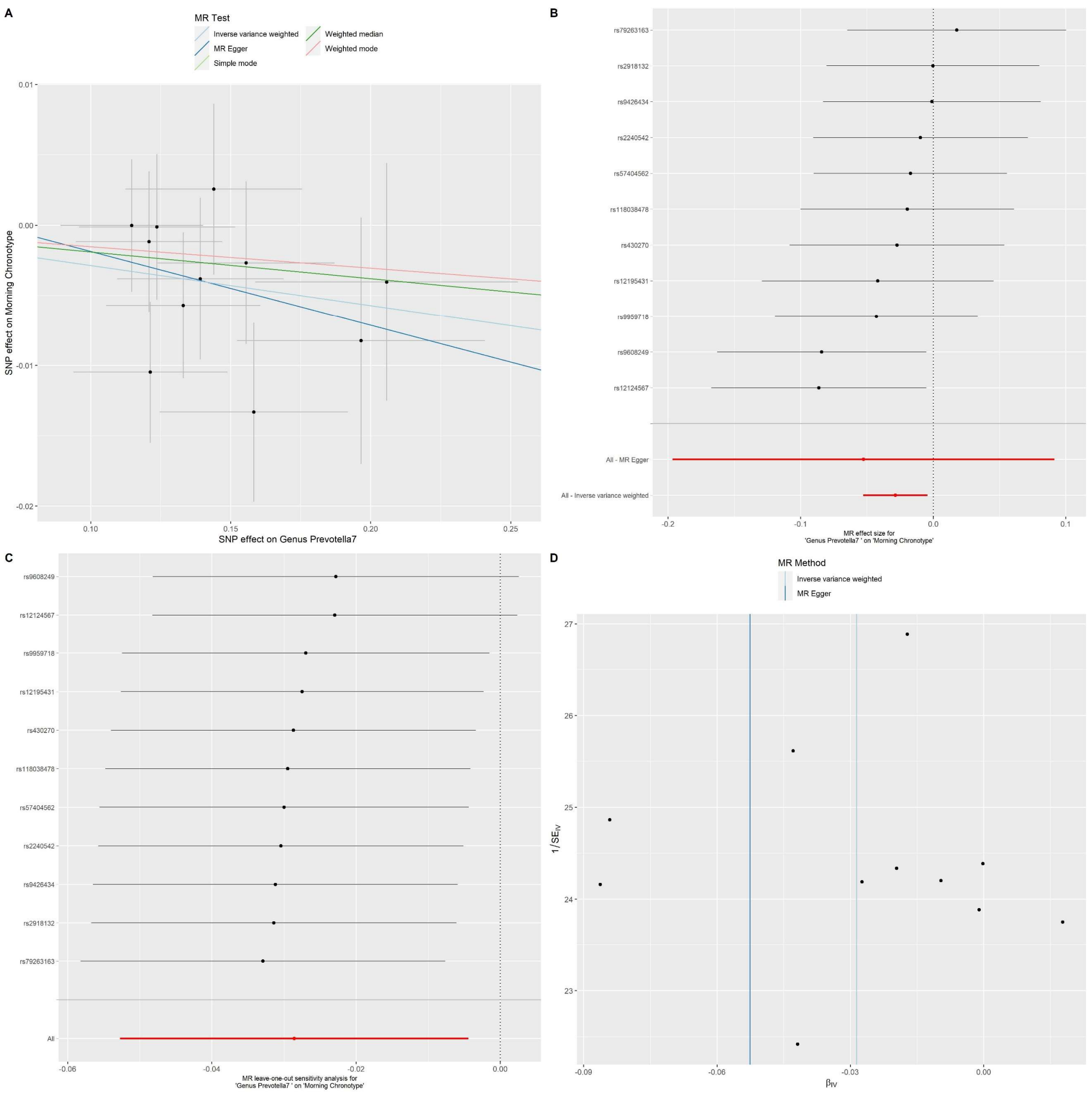

Figure S32. Mendelian randomization plots for the relationship of Genus *Prevotella7* with morning chronotype

Note: A, Scatterplot of SNP effects on morning chronotype with the slope of each line corresponding to estimated MR effect (Inverse Variance Weighted, Weight Median, MR-Egger, Weighted Mode, and Simple Mode methods); B, Forest plot of individual and combined SNP MR-estimated effects sizes for relative morning chronotype; C, The leave-one-out plot visualized how the causal estimates (point with horizontal line) for the effect of morning chronotype on morning chronotype were influenced by the removal of single variant; D, Funnel plot assessing heterogeneity. Blue line represents the inverse-variance weighted estimate, and dark blue line represents the MR-Egger estimate.

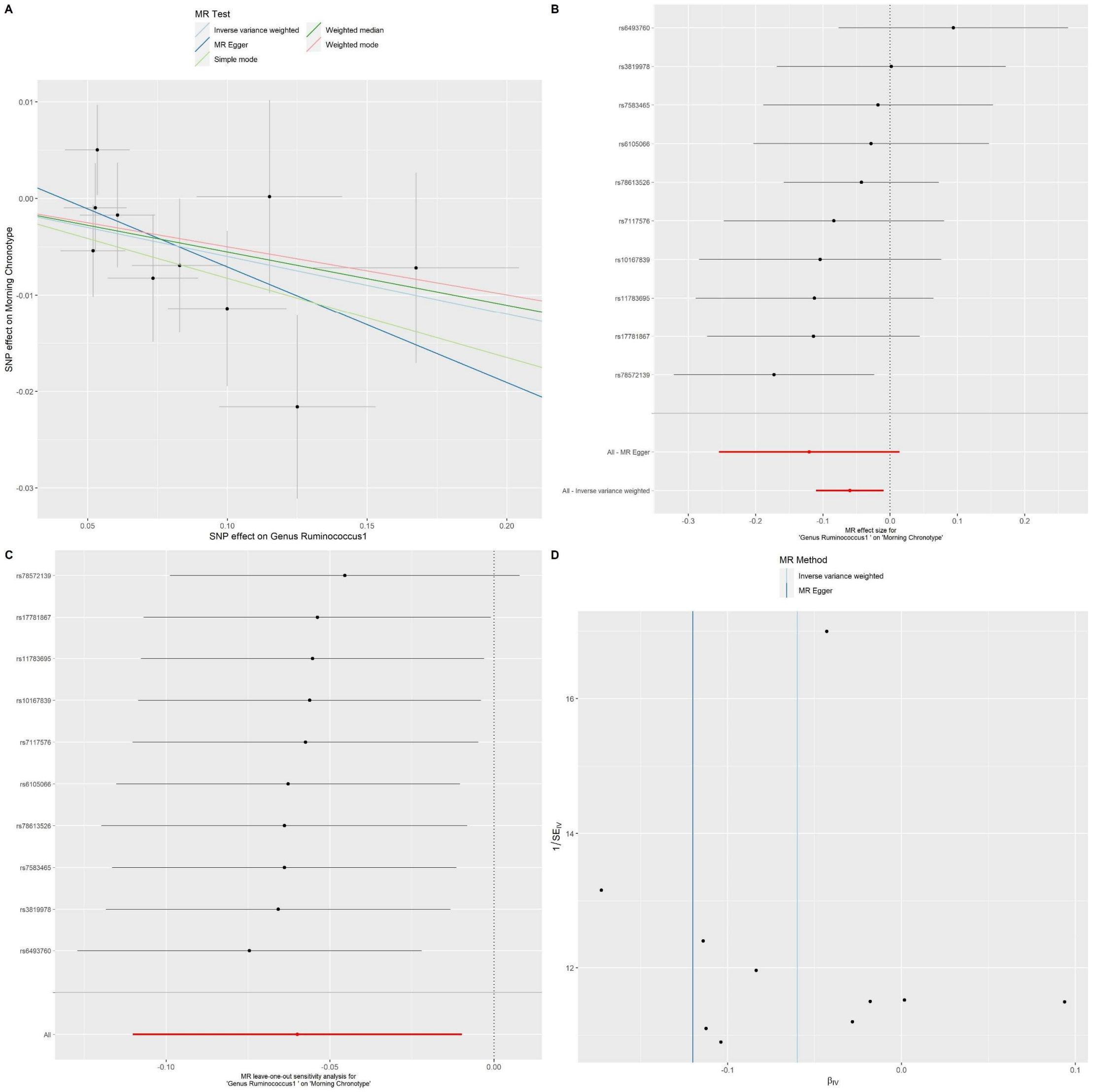

Figure S33. Mendelian randomization plots for the relationship of Genus *Ruminococcus1* with morning chronotype

Note: A, Scatterplot of SNP effects on morning chronotype with the slope of each line corresponding to estimated MR effect (Inverse Variance Weighted, Weight Median, MR-Egger, Weighted Mode, and Simple Mode methods); B, Forest plot of individual and combined SNP MR-estimated effects sizes for relative morning chronotype; C, The leave-one-out plot visualized how the causal estimates (point with horizontal line) for the effect of morning chronotype on morning chronotype were influenced by the removal of single variant; D, Funnel plot assessing heterogeneity. Blue line represents the inverse-variance weighted estimate, and dark blue line represents the MR-Egger estimate.

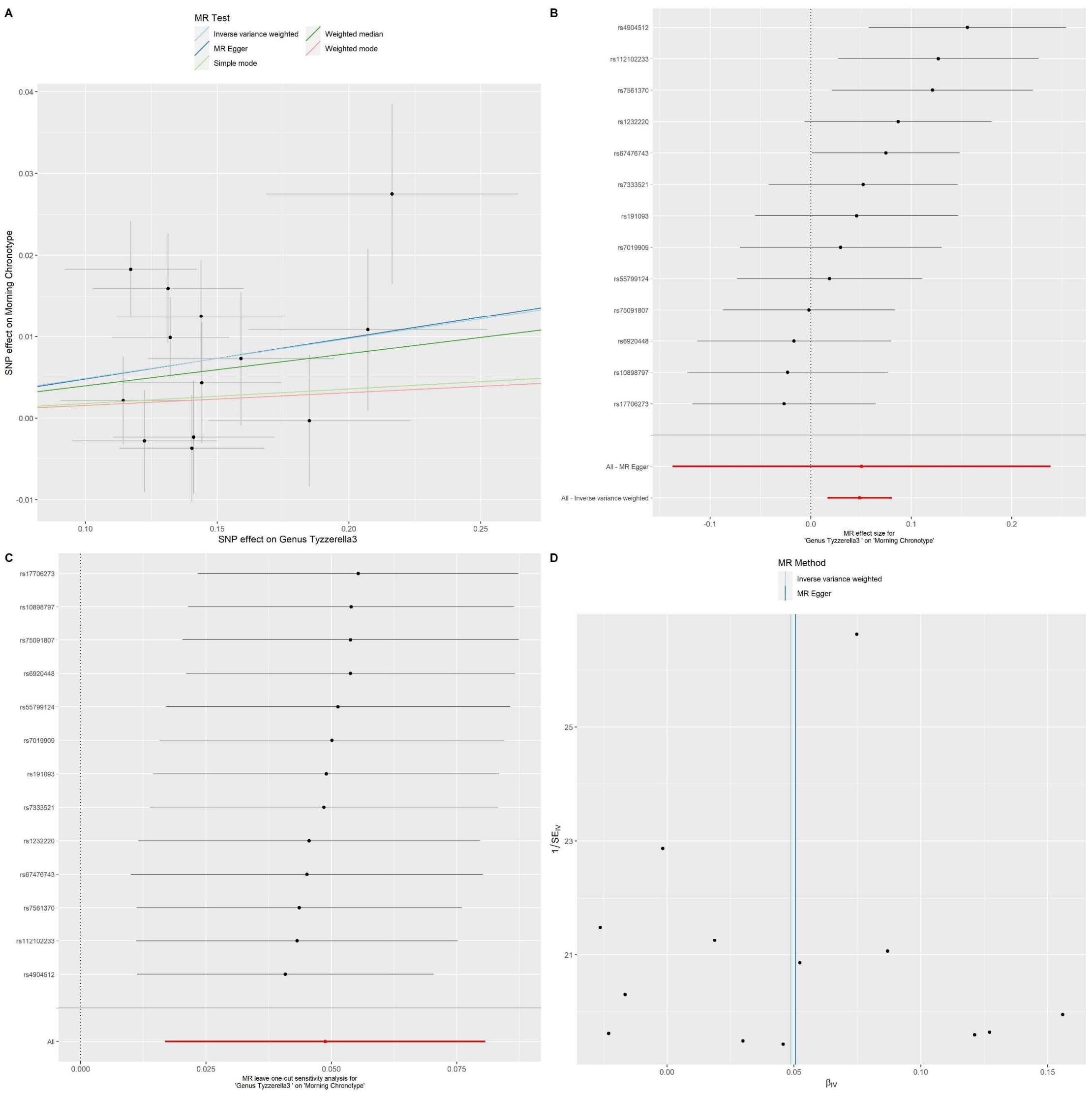

Figure S34. Mendelian randomization plots for the relationship of Genus *Tyzzerella3* with morning chronotype

Note: A, Scatterplot of SNP effects on morning chronotype with the slope of each line corresponding to estimated MR effect (Inverse Variance Weighted, Weight Median, MR-Egger, Weighted Mode, and Simple Mode methods); B, Forest plot of individual and combined SNP MR-estimated effects sizes for relative morning chronotype; C, The leave-one-out plot visualized how the causal estimates (point with horizontal line) for the effect of morning chronotype on morning chronotype were influenced by the removal of single variant; D, Funnel plot assessing heterogeneity. Blue line represents the inverse-variance weighted estimate, and dark blue line represents the MR-Egger estimate.

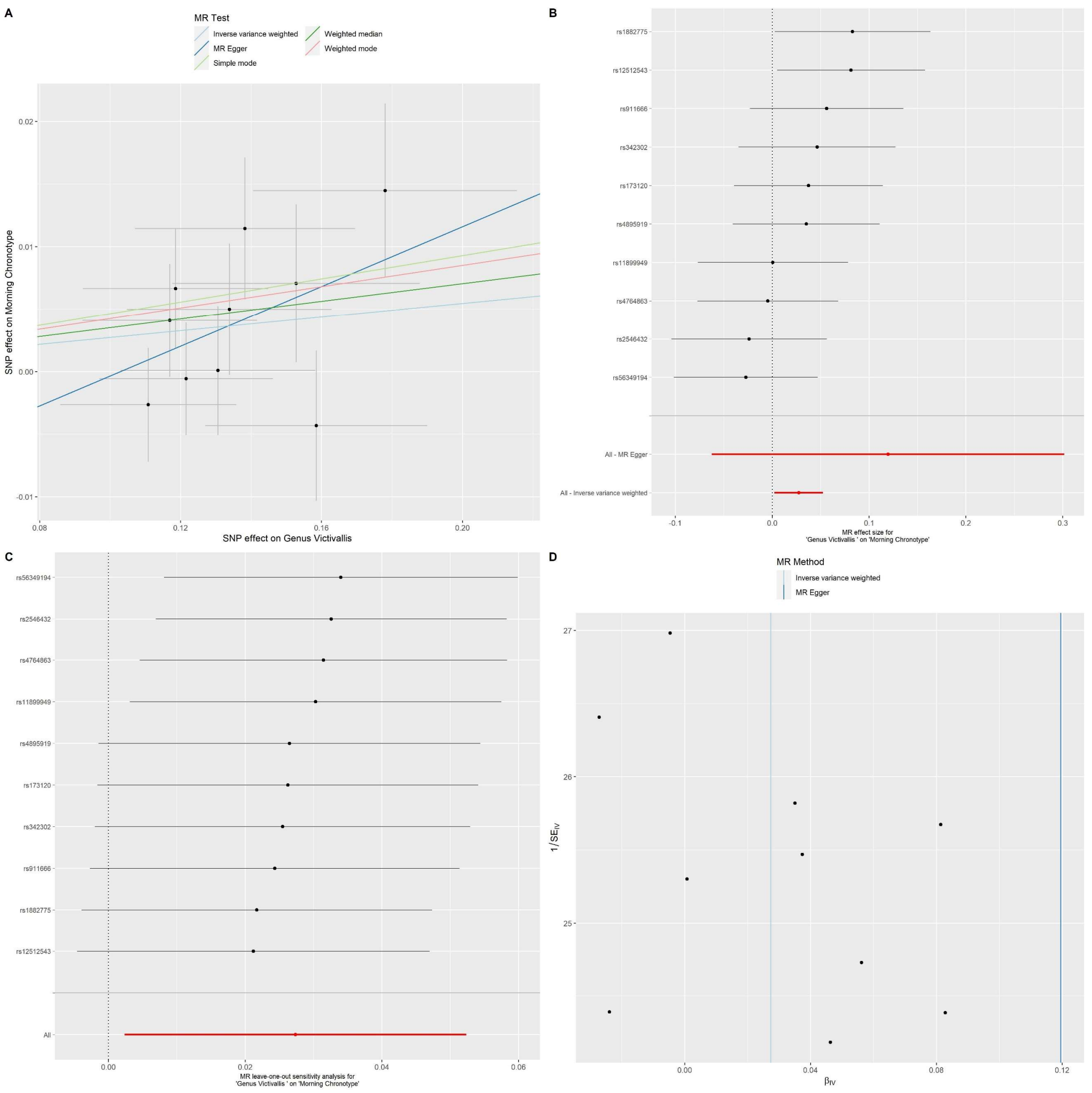

Figure S35. Mendelian randomization plots for the relationship of Genus *Victivallis* with morning chronotype

Note: A, Scatterplot of SNP effects on morning chronotype with the slope of each line corresponding to estimated MR effect (Inverse Variance Weighted, Weight Median, MR-Egger, Weighted Mode, and Simple Mode methods); B, Forest plot of individual and combined SNP MR-estimated effects sizes for relative morning chronotype; C, The leave-one-out plot visualized how the causal estimates (point with horizontal line) for the effect of morning chronotype on morning chronotype were influenced by the removal of single variant; D, Funnel plot assessing heterogeneity. Blue line represents the inverse-variance weighted estimate, and dark blue line represents the MR-Egger estimate.

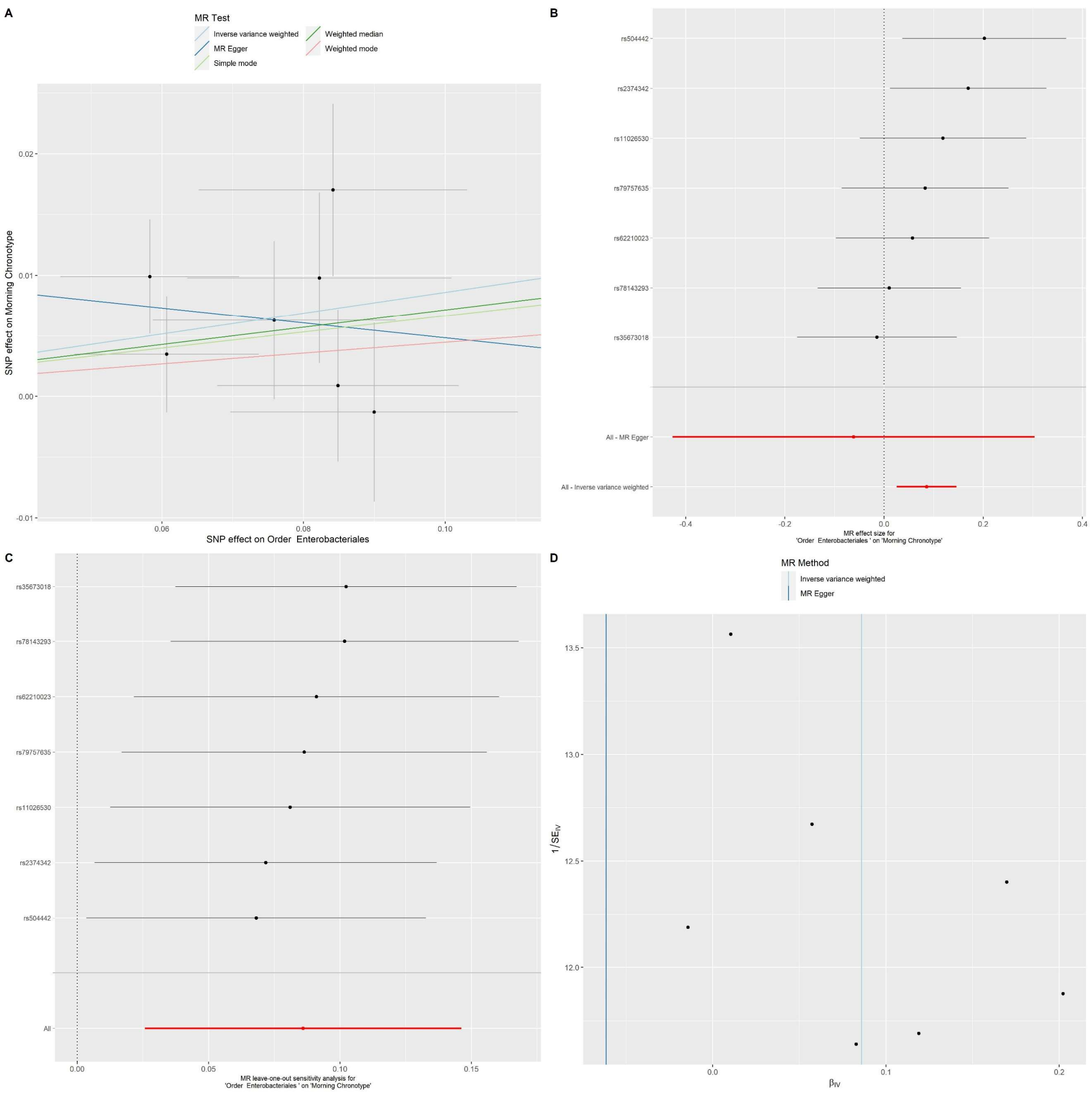

Figure S36. Mendelian randomization plots for the relationship of Order *Enterobacteriales* with morning chronotype

Note: A, Scatterplot of SNP effects on morning chronotype with the slope of each line corresponding to estimated MR effect (Inverse Variance Weighted, Weight Median, MR-Egger, Weighted Mode, and Simple Mode methods); B, Forest plot of individual and combined SNP MR-estimated effects sizes for relative morning chronotype; C, The leave-one-out plot visualized how the causal estimates (point with horizontal line) for the effect of morning chronotype on morning chronotype were influenced by the removal of single variant; D, Funnel plot assessing heterogeneity. Blue line represents the inverse-variance weighted estimate, and dark blue line represents the MR-Egger estimate.

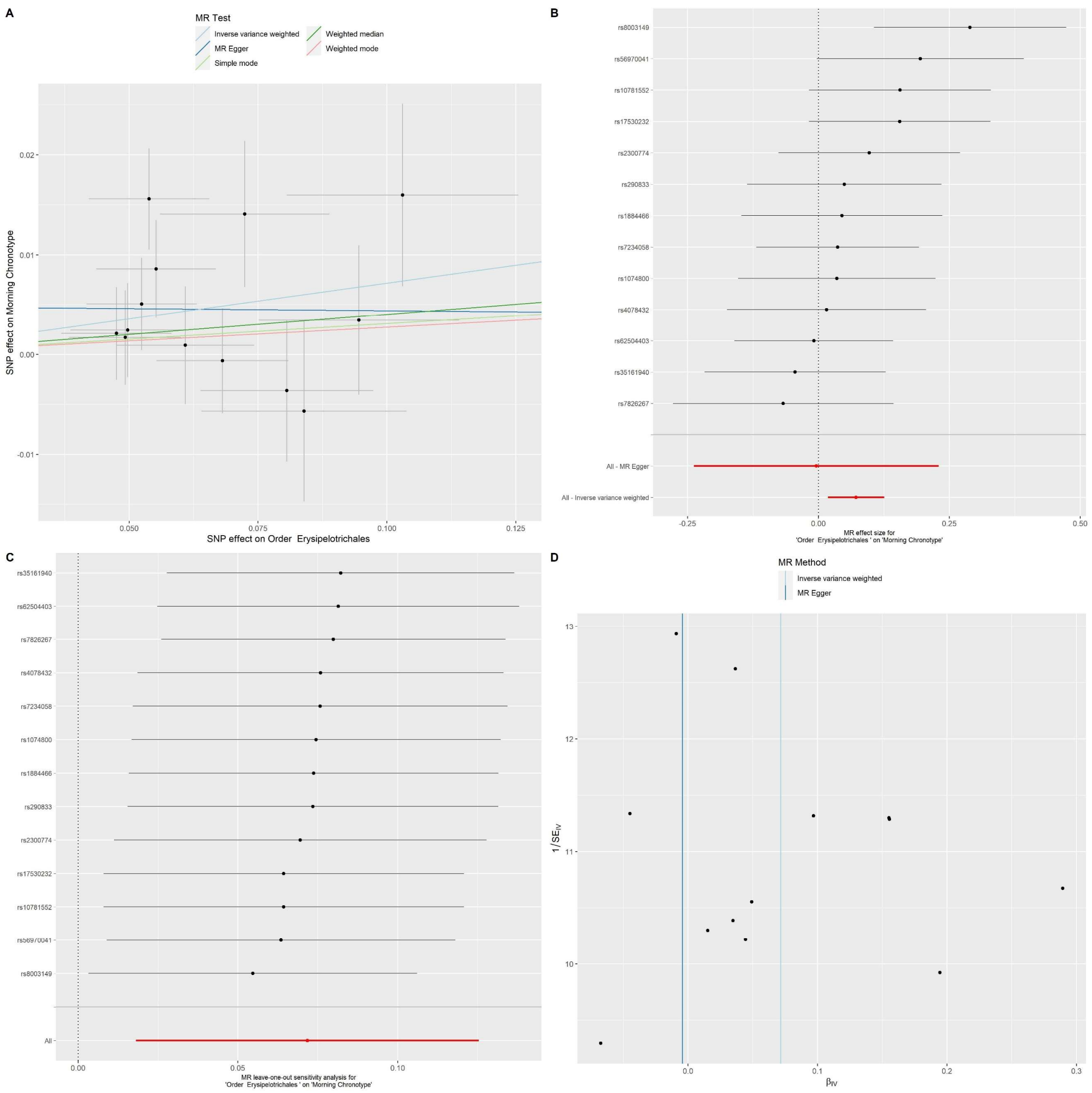

Figure S37. Mendelian randomization plots for the relationship of Order *Erysipelotrichales* with morning chronotype

Note: A, Scatterplot of SNP effects on morning chronotype with the slope of each line corresponding to estimated MR effect (Inverse Variance Weighted, Weight Median, MR-Egger, Weighted Mode, and Simple Mode methods); B, Forest plot of individual and combined SNP MR-estimated effects sizes for relative morning chronotype; C, The leave-one-out plot visualized how the causal estimates (point with horizontal line) for the effect of morning chronotype on morning chronotype were influenced by the removal of single variant; D, Funnel plot assessing heterogeneity. Blue line represents the inverse-variance weighted estimate, and dark blue line represents the MR-Egger estimate.

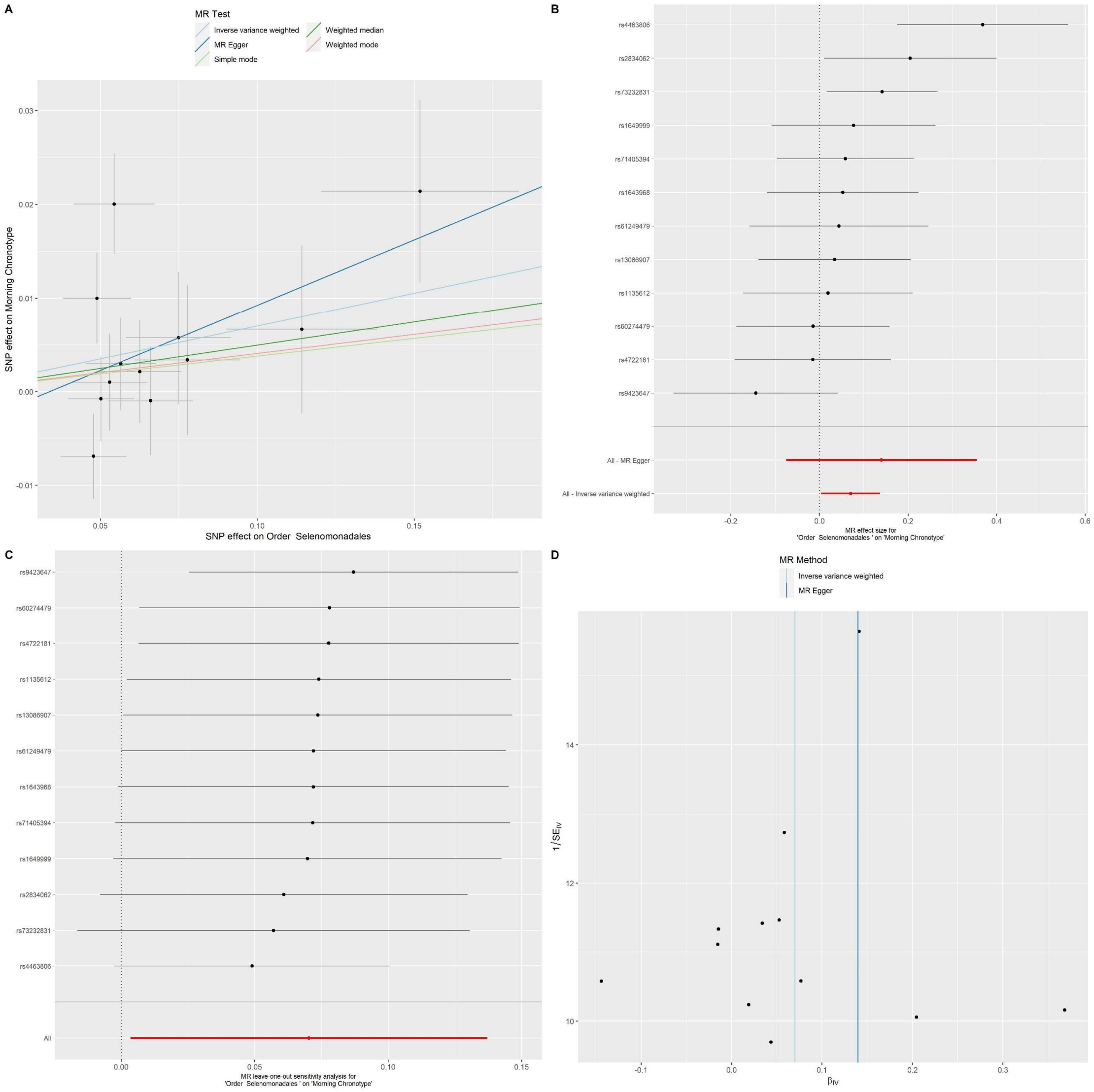

Figure S38. Mendelian randomization plots for the relationship of Order *Selenomonadales* with morning chronotype

Note: A, Scatterplot of SNP effects on morning chronotype with the slope of each line corresponding to estimated MR effect (Inverse Variance Weighted, Weight Median, MR-Egger, Weighted Mode, and Simple Mode methods); B, Forest plot of individual and combined SNP MR-estimated effects sizes for relative morning chronotype; C, The leave-one-out plot visualized how the causal estimates (point with horizontal line) for the effect of morning chronotype on morning chronotype were influenced by the removal of single variant; D, Funnel plot assessing heterogeneity. Blue line represents the inverse-variance weighted estimate, and dark blue line represents the MR-Egger estimate.
